# A novel predictor of ACE2-binding ability among betacoronaviruses

**DOI:** 10.1093/emph/eoab032

**Published:** 2021-10-13

**Authors:** Jamie D Dixson, Rajeev K Azad

**Affiliations:** 1 Department of Biological Sciences and BioDiscovery Institute, University of North Texas, Denton, TX 76203, USA; 2 Department of Mathematics, University of North Texas, Denton, TX 76203, USA

**Keywords:** SARS-CoV-2, betacoronaviruses, virus evolution, biosurveillance

## Abstract

**Background:**

Coronavirus disease 2019 (COVID-19), caused by severe acute respiratory syndrome coronavirus 2 (SARS-CoV-2), has resulted in ~4.8 million deaths worldwide as of this writing. Almost all conceivable aspects of SARS-CoV-2 have been explored since the virus began spreading in the human population. Despite numerous proposals, it is still unclear how and when the virus gained the ability to efficiently bind to and infect human cells. In an effort to understand the evolution of receptor binding domain (RBD) of the spike protein of SARS-CoV-2, and specifically, how the ability of RBD to bind to angiotensin-converting enzyme 2 receptor (ACE2) of humans evolved in coronaviruses, we have applied an alignment-free technique to infer functional relatedness among betacoronaviruses. This technique, concurrently being optimized for identifying novel prions, was adapted to gain new insights into coronavirus evolution, specifically in the context of the ongoing COVID-19 pandemic. Novel methods for predicting the capacity for coronaviruses, in general, to infect human cells are urgently needed.

**Methodology:**

proposed method utilizes physicochemical properties of amino acids to develop fully dynamic waveform representations of proteins that encode both the amino acid content and the context of amino acids. These waveforms are then subjected to dynamic time warping (DTW) and distance evaluation to develop a distance metric that is relatively less sensitive to variation in sequence length and primary amino acid composition.

**Results and Conclusions:**

Using our proposed method, we show that in contrast to alignment-based maximum likelihood (ML) and neighbor-joining (NJ) phylogenetic analyses, all bat betacoronavirus spike protein RBDs known to bind to the ACE2 receptor are found within a single physicochemical cluster. Further, other RBDs within that cluster are from pangolin coronaviruses, two of which have already been shown to bind to ACE2 while the others are suspected, yet unverified ACE2 binding domains. This finding is important because both severe acute respiratory syndrome coronavirus (SARS-CoV) and SARS-CoV-2 use the host ACE2 receptor for cell entry. Surveillance for coronaviruses belonging to this cluster could potentially guide efforts to stifle or curtail potential and/or early zoonotic outbreaks with their associated deaths and financial devastation.

**Lay Summary:**

Robust methods for predicting human ACE2 receptor binding by the spike protein of coronaviruses are needed for the early detection of zoonotic coronaviruses and biosurveillance to prevent future outbreaks. Here we present a new waveform-based approach that utilizes the physicochemical properties of amino acids to determine the propensity of betacoronaviruses to infect humans. Comparison with the established phylogenetic methods demonstrates the usefulness of this new approach in the biosurveillance of coronaviruses.

## INTRODUCTION

Coronaviruses are a diverse group of RNA viruses of the family Coronaviridae. There are currently four recognized genera within this family, namely, *Alphacoronavirus*, *Betacoronavirus*, *Gammacoronavirus* and *Deltacoronavirus* [1]. Of these, members of the *Alphacoronavirus* and *Betacoronavirus* are known to primarily infect mammals including humans [1, 2]. There are seven coronaviruses known to infect humans. These include SARS-CoV, SARS-CoV-2, Middle East respiratory syndrome-related coronavirus (MERS-CoV), HCoV-NL63 and HCoV-HKU1 that were identified after the SARS-CoV epidemic of 2002–03, and HCoV-OC43 and HCoV-229E that were identified in samples collected in the 1960s. Of these, all but HCoV-NL63 and HCoV-229E are betacoronaviruses [3–11].

In the past 20 years, three of the seven known human coronaviruses have emerged, presumptively through zoonoses, as deadly human pathogens. These include *Betacoronavirus* SARS-CoV that in November 2002 began circulating with human-to-human transmission in China [12], *Betacoronavirus* MERS-CoV that in 2012 made the leap to humans in the middle-east with human-to-human transmission limited primarily to healthcare settings [7, 13], and a second *Betacoronavirus* SARS-CoV-2 that began circulating in the human population of Guangdong Province, China in late 2019 [14]. Of grave concern is that both SARS-CoV and SARS-CoV-2, the two most contagious and therefore deadly human betacoronaviruses, have emerged recently and as shown in Table  1, both use the ACE2 receptor for entry into human cells [15, 16]. Of even greater concern is that these viruses may have converged upon efficient human ACE2 usage since the ML phylogenetic analyses of the region of the spike protein responsible for binding of the ACE2 receptor indicates considerable divergence in the sequences of these domains between SARS-CoV and SARS-CoV-2 [17]. Therefore, prior to the placement of SARS-CoV-2 into the second clade of ACE2-binding betacoronaviruses, the scientific community might have erroneously assumed that the clade was less of a zoonotic concern. For this reason, it is imperative that efficient and cost-effective surveillance techniques be developed which allow for the identification of betacoronaviruses with the ability to bind ACE2 or the propensity to converge upon human ACE2 usage. Herein we present one such method.

**Table 1.** Betacoronaviruses known to bind the ACE2 receptor

Virus	Host	Acc. Num.	References
SARS-CoV	Human	P59594	[18]^**a**^
SARS-CoV-2	Human	P0DTC2	[19]^a^
GXP2V	Pangolin	A0A6G9KP06	[20]^a^
MP789	Pangolin	A0A6M3G9R1	[21]^a^
RS7327	Bat	A0A2D1PXC0	[16]^a^
YN2018B	Bat	A0A4Y6GL47	[15]^a^
Rs9401	Bat	A0A2D1PXD5	[15]^b^
WIV16	Bat	A0A0U2IWM2	[22]^a^
Rs4874	Bat	A0A2D1PX97	[15]^b^
Rs3367	Bat	U5WHZ7	[15]^b^
WIV1	Bat	U5WI05	[23]^a^
Rs4084	Bat	A0A2D1PX29	[16]^a^
RsSHC014	Bat	U5WLK5	[24]^a^
Rs4231	Bat	A0A2D1PXA9	[16]^a^
RaTG13	Bat	A0A6B9WHD3	[25]^a^

Cited evidence for ACE2 usage based either on demonstration of binding^a^ or bioinformatic prediction of binding^b^. In cases where the prediction was made prior to demonstration, the citation for the demonstration is shown. Others have hypothesized that the additional pangolin coronaviruses included in our analysis also bind ACE2, however, this has not yet been investigated.

The question of the origin of any organism, protein, or gene is a question of both homology and mapping of that homology. Current phylogenetic analyses mostly involve comparison and quantification of similarity among biomolecular entities, mainly at the nucleotide or protein primary sequence level. Arguably the most objective approach to classify genetic entities is using quantifiable traits even when that quantification is boolean in nature [26]. This is the reason for the popularity of nucleotide and amino acid sequence-based approaches for placing a value on the degree of similarity and inferring relatedness based on that similarity. Classification is simple and intuitive once alignment of nucleotide or amino acid sequences is achieved. However, in cases where sequences are evolving rapidly, the similarity between sequences may be low despite conservation of function. This may render sequences unalignable or yield an alignment of too low of a score to be deemed significant. In such cases, inferring homology of rapidly evolving sequences based on alignment may be inconclusive or incorrect. This has necessitated the development of alternative approaches to infer relationships whether phylogenetic or functional, especially when the evolutionary distance between biological entities is large or evolutionary rate is high. Lolkema and Slotboom [27] took a step forward in that direction when they examined protein sequences from the aspect of shared properties rather than corresponding sequence identity and made the statement that ‘three-dimensional structure is quite tolerant of changes at the amino acid level’. This engenders the need for approaches to the determination of homology, which do not strictly rely upon primary sequence alignment. However, it also fails to include the related but distinct conundrum that a single amino acid substitution/insertion/deletion may in some cases completely change the functional niche filled by a protein and thus may represent a level of functional evolution or divergence that cannot otherwise be inferred based on alignment-based techniques.

When sequence identity as determined by primary sequence alignment is low and yet functional conservation is obvious, homology determination becomes difficult, that is, many such cases fall into the twilight zone of <30% identity among homologs where ambiguity rules [28]. Of even further complication in determining homology is the midnight zone of the twilight zone (≤10% identity) where homology and random chance have an almost equal probability of resulting in observed functional similarity among protein structures [28, 29]. Alignment-free techniques like the one presented herein attempt to reach into the twilight zone of sequence identity and resolve function/structure-based relatedness instead of or in support of weak sequence identity-based determinations. This is important considering the vast amounts of biomolecular data where relatedness has been difficult to accurately determine [29]. It should be emphasized that the twilight zone becomes a limiting factor when evolutionary distance is high, not necessarily temporal distance. In other words, two sequences or even very small fragments of a gene may diverge very rapidly in a short period of time under certain circumstances and thus temporally speaking enter the twilight zone rapidly.

In the early years of phylogenetics, perhaps when biomolecular sequences were not abundant, homology was predominately determined using traits expressed at the macro-morphological scale. This practice continues even today, albeit in a limited scope and most notably in cases where molecular data are not available or collection is not practical such as in the analysis of fossils [30] and fish genetics where large numbers of individuals from closely related taxa are regularly defined by both morphological and meristic metrics [31]. However, morphological traits to characterize taxa need not only be macro-morphological. In other words, the conformation of a protein, if it can be mathematically described in part or whole is also a morphometric trait and the count of transmembrane regions, domains, or residues if observable or predictable can also be meristic despite the need for molecular-level resolution for observation [26]. Lolkema and Slotboom [27] recognized this and proposed that the hydropathy profile of proteins could be used to find distant homologs. Eight years after that proposal, hydropathy profiles were successfully used in a phylogenetic inquiry. In that study, each amino acid was categorically classified as internal (I), external (E), or ambivalent (A) based on its hydrophobicity (HP) [32]. Those transformed strings representing the hydrophobic category of each amino acid were then used for pairwise input into the Lempel and Ziv algorithm whose complexity metric was used to derive a measure of distance between proteins [32, 33].

Rather than inferring the substitution of amino acids based on probabilistic models derived from the alignments of well-understood protein sequences, which underpin most scoring matrices commonly used for sequence alignment, the inference by the method presented herein is based solely on the assumption of conservation of structure/function within orthologous protein domains. Such structural conservation likely varies spatially within a protein and therefore our alignment-free method, not constrained by genomic segment contiguity relied upon by alignment-based methods, is able to account for such conservation at the structural/functional level and thus could resolve the twilight zone homology, where the alignment-based methods relying on genomic segment contiguity and probabilistic matrices become progressively less reliable.

The physicochemical properties that we chose to mathematically represent the underlying structure of the RBDs of coronaviruses were HP and molecular weight (MW). While HP is well established as the premier factor with regards to the formation of stable protein–protein interaction [34–36], MW as an indicator of accessible surface area and shape is also fundamental to such interactions [34, 35]. Only when an optimal combination of spatial HP and shape exists, two proteins can form a stable complex of complementary structure. Therefore, in the absence of complementarity with regards to HP and shape, other physicochemical properties that contribute to the specificity of protein–protein interactions may be of limited effect on the stability/specificity of the protein–protein complex [35]. For this reason, we chose both HP and MW as contributing factors in the derivation of the waveforms used in this study.

We therefore build upon the use of HP by Lolkema and Slotboom [27] by including the MW of each amino acid as well in order to derive a representative profile that can then be used in functional cluster analyses of the spike protein RBD of betacoronaviruses. It should be noted that although our method has some similarities of concept with that of the Liu and Wang method [32], the genesis of our method did not start with their research. Our method was conceived and evolved independently, in our ongoing attempt to classify novel proteins belonging to functional groups with little to almost no sequence-level identity and high levels of variation in sequence length, namely, the prions. As previously stated, one of the most profound differences between our method and the Liu and Wang approach is that our method uses not only the HP of each amino acid but also the MW to derive a fully dynamic non-categorical profile for each protein, based on the primary amino acid sequence. Furthermore, and as expanded upon later, our approach utilizes DTW to derive a distance measure that can subsequently be used in clustering analysis and/or potentially phylogenetic reconstruction. Furthermore, our method does not necessarily assign the same index value to the same amino acid at two different positions within a protein because the residues that flank each amino acid have an effect on the value assigned to that amino acid. Therefore, our method allows for the construction of an indexed profile of a protein as encoded by a serial string of amino acids, accounting for both, content and context. The method of Liu and Wang was, however, predominately based on the content [32].

## METHODS

### Waveform conversion

Primary amino acid sequences can be converted into vector representations according to the physicochemical properties of each individual residue. In doing so it becomes possible to visualize polypeptide sequences as waveforms (the terms wave, waveform and vector are used interchangeably henceforth). These are similar in concept to time series data except that periodicity is based on a serial representation of residues rather than observations at sampling times. The techniques employed are, therefore, conceptually similar to those used in the analysis of time series data such as speech recognition profiles.

A complete list of the sequences used in our analyses can be found in Supplementary Table S1. For each of the coronaviruses examined, only the sequence of the RBD of the spike protein was used in our analysis. Unless noted otherwise, all analyses were performed on a single computer with an Intel^®^Core™i5-7200u processor with 8GB of RAM. Each RBD sequence was converted into a vector according to the algorithm described below. The algorithm uses the values for residue MW and HP to compute residual value *V* (Equation (1); Table  2). The values for residue MW and HP are available at https://www.sigmaaldrich.com/life-science/metabolomics/learning-center/amino-acid-reference-chart.html#3 (2 November 2021, date last accessed) and originally published by Monera et al. [37].

(1) V=(R/G)*H

Where *V* is the residue value; *R* is the residue MW; *G* is the largest MW of all 20 amino acids (186.22); and *H* is the residue HP index.

**Table 2.** Values (V) representing the combined properties of HP and MW for pairs of amino acids.

Residue	**G**	A	L	M	F	W	K	Q	E	S	P	V	I	C	Y	H	R	N	D	T
**G**	0.306																			
**A**	7.978	15.650																		
**L**	30.537	38.208	60.767																	
**M**	26.221	33.893	56.452	52.136																
**F**	38.486	46.157	68.716	64.400	76.665															
**W**	48.653	56.325	78.883	74.568	86.832	97.000														
**K**	−7.763	−0.091	22.468	18.152	30.417	40.584	−15.832													
**Q**	−3.287	4.385	26.943	22.628	34.892	45.060	−11.356	−6.881												
**E**	2.927	10.598	33.157	28.842	41.106	51.274	−5.142	−0.667	5.547											
**S**	−1.016	6.656	29.214	24.899	37.163	47.331	−9.085	−4.609	1.605	−2.338										
**P**	−11.842	−4.170	18.388	14.073	26.337	36.505	−19.911	−15.436	−9.222	−13.164	−23.991									
**V**	20.382	28.053	50.612	46.297	58.561	68.729	12.313	16.788	23.002	19.059	8.233	40.457								
**I**	30.233	37.905	60.463	56.148	68.412	78.580	22.164	26.639	32.853	28.911	18.084	50.308	60.159							
**C**	13.724	21.396	43.954	39.639	51.903	62.071	5.655	10.131	16.344	12.402	1.576	33.799	43.651	27.142						
**Y**	21.622	29.294	51.852	47.537	59.801	69.969	13.553	18.029	24.242	20.300	9.474	41.697	51.548	35.040	42.938					
**H**	−11.262	−3.590	18.969	14.653	26.917	37.085	−19.331	−14.855	−8.641	−12.584	−23.410	8.814	18.665	2.156	10.054	−22.830				
**R**	−5.718	1.954	24.512	20.197	32.461	42.629	−13.787	−9.312	−3.098	−7.040	−17.866	14.357	24.209	7.700	15.598	−17.286	−11.742			
**N**	−8.426	−0.754	21.805	17.489	29.754	39.921	−16.495	−12.019	−5.805	−9.748	−20.574	11.650	21.501	4.992	12.890	−19.994	−14.450	−17.158		
**D**	−16.843	−9.171	13.388	9.072	21.336	31.504	−24.912	−20.436	−14.222	−18.165	−28.991	3.233	13.084	−3.425	4.473	−28.411	−22.867	−25.575	−33.992	
**T**	3.683	11.354	33.913	29.597	41.862	52.029	−4.387	0.089	6.303	2.360	−8.466	23.758	33.609	17.100	24.998	−7.886	−2.342	−5.050	−13.467	7.059

The values on the diagonal were calculated using Equation (1) and the lower triangle values represent the means of the individual values for a given pair of residues.

The values in Table  2 were used to calculate a vector representation of each RBD. This conversion from primary amino acid sequence to the vector representation was carried out by calculating three peak values for each residue as shown in Table  3. The two peaks that flank each residue are called ‘phantom’ peaks and the vector representation values for the three peaks were calculated as described below. The intrinsic value for the left flanking peak is the value of *V* for the residue of interest and the extrinsic value was obtained as the mean of the *V* value for the residue of interest and *V* value for the left flanking residue (Table  3). The vector representation value for the left flanking peak was then obtained as the mean of the intrinsic and extrinsic values. Similarly, the intrinsic value for the right flanking peak is the value of *V* for the residue of interest and the extrinsic value was obtained as the mean of the *V* value for the residue of interest and *V* value for the right flanking residue (Table  3). The vector representation value for the right flanking peak was obtained as the mean of these intrinsic and extrinsic values. The intrinsic value for the central peak is the value of *V* for the residue of interest and the extrinsic value is the cumulative extrinsic values from the flanking peaks. The vector representation value for the central peak was obtained as the mean of these intrinsic and extrinsic values. In order to start each wave, it was assumed that an additional glycine residue occurred prior to the start of the sequence. In order to end each wave, it was assumed that an additional residue identical to the last residue of the actual sequence occurred after the last residue. These assumptions were in effect inconsequential and were only used as a convenient way to approximate the beginning and end of the wave. In addition, we also analyzed single-peak waveforms whereby each peak for a residue corresponded to the mean of intrinsic (*V* value of the residue, Table  2) and external forces (sum of both flanking residue values, Table  2). We developed an R-Script for our analyses in which both the single-peak and three-peak algorithms were implemented. The R-Script is available on GitHub at https://github.com/JamberFX/Mol-WtandHydrophobicityDTW (Accessed 02 November 2021).

**Table 3.** Example conversion from primary amino acid sequence to vector (waveform) representation of a short amino acid sequence.

Original Sequence:		G			A			T	
Profile:	Phantom	G	Phantom	Phantom	A	Phantom	Phantom	T	Phantom
Internal Values:	0.306 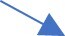	0.306	0.306	15.650	15.650	15.650	7.059	7.059	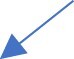 7.059
External Values:	–	0.306	7.978	7.978 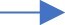	7.978	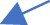 11.354	11.354 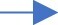	11.354	–
	–	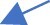 0.978	–	–	11.354	–	–	7.059	–
Vector:	0.306	4.295	4.142	11.814	17.491	13.502	9.207	12.736	7.059

The vector value for phantom peaks are means and the vector values for actual residues are one-half of the sum of all internal and external values for that residue.

### Dynamic time warping and hierarchical clustering

RBD polypeptide sequences were analyzed by performing direct Euclidean and cosine comparisons using the ape, dtw, proxy and TreeTools libraries in R [38–43]. Three-peak and single-peak waveforms as described above were evaluated. RBD polypeptide sequences were also evaluated using DTW. For DTW, only Euclidean distance was used because with univariate vectors, such as those used in this study, warping does not generally vary based on distance metric and also because if we had developed a similarity matrix using DTW with cosine distance, the distances would have all been zero, or near zero, due to the calculation of cosine distance. For both direct and DTW analyses, the resulting distance matrices for the full suite of sequences considered were subjected to hierarchical clustering using both unweighted pair group method with arithemetic meand (UPGMA) and NJ as implemented in the hclust and nj functions of core R [41].

In addition to the waveform-based, alignment-free approach, we reconstructed phylogenetic trees for the amino acid sequences using alignment-based ML and NJ protocols. Amino acid sequences were used in our alignment-based analysis because the results from such analyses are directly comparable to our DTW-based approach which uses the physicochemical properties of those amino acids to derive distance and topological relationships. For the alignment-based analyses, multiple sequence alignments were performed using ClustalW version 2.1 as implemented in Jalview 2.11.1.3 with a gap initiation penalty of 10, a gap extension penalty of 0.2, and a Gonnet series scoring matrix (default) [44, 45]. ML tree reconstruction was performed using the PhyML webserver available at http://www.atgc-montpellier.fr/phyml/ (Accessed 02 November 2021), with automatic substitution model selection (default) and 1000 bootstrap steps [46]. NJ tree reconstruction was performed using MEGA-X version 10.1.8 with the Jones-Taylor-Thornton (JTT) substitution model with the gamma rate among sites set to 1.0 (default) and 1000 bootstrap steps [47].

### Dendrogram congruence evaluation

Three methods of determining the congruence among dendrograms were used. These included visual examination of tanglegrams, evaluation of the number of nodes with the exact same list of terminal taxa (aka common nodes) and calculation of the Robinson–Foulds Metric [48]. Tanglegram comparisons of full trees were performed using the phytools package in R [49], common node calculations were performed using the dendextend package in R [50] and Robinson–Foulds metrics were calculated using the TreeDist package in R [51]. Individual trees included herein were prepared for publication using FigTree v.1.4.3 [52] and TreeGraph 2.15 [53].

### Dynamic time warping one-to-one waveform comparison

To compare the waveform representation of RBD sequences from different coronaviruses including SARS-CoV-2, we used the Warping Correspondence function in Mathematica version 11.3.0.0 [54] with a Euclidean distance function. The Euclidean distance between the SARS-CoV-2’s RBD waveform and each of P59594 (SARSCoV), A0A6B9WHD3 (BtCoV/RaTG13), A0A6G9KP06 (PCoV_GXP2V), A0A6G6A2R8 (PCoV_GXP1E), and A0A6M3G9R1 (PCoV_GD_MP789) was calculated along the full length of the waveform using a window size of 10 and an offset of 1. It is important to note that there is no universal reference when using DTW. Therefore, each pair of sequences may experience varied levels and locations of *X*-axis warping. In other words, differences indicated by peaks in the plots of these waveforms may not be directly comparable across multiple waveforms unlike multiple alignment-based methods.

## RESULTS

**Figure 1.**
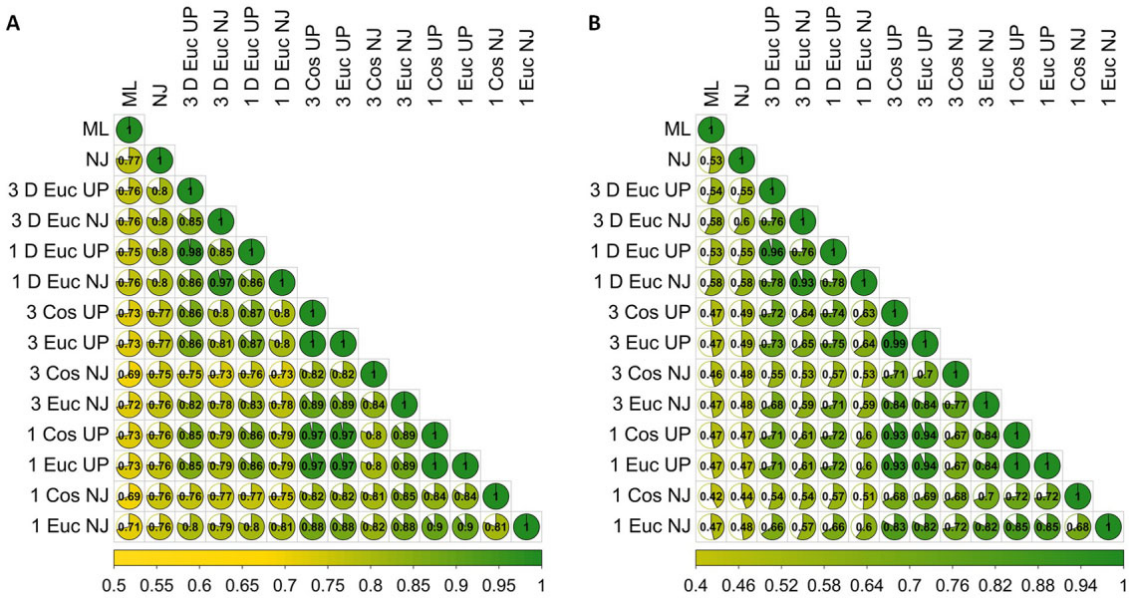
(A) Proportion of common nodes (nodes with the exact same list of terminal taxa) depicted in the dendrogram comparisons from all methods used to resolve the relationships among spike protein receptor-binding domains. (B) Inverse Normalized Robinson–Foulds Metric for the dendrogram comparisons of all methods used to resolve the relationships among spike protein receptor-binding domains. MW and HP were used as the physicochemical properties for all methods as described in the methods section. The first two methods shown were standard alignment-based ML and alignment-based NJ analyses of the amino acid sequences. The remaining 12 analyses shown used waveforms as described in the Methods section. Among those methods, the first part of the label for each method indicates whether a three-peak or single-peak waveform was encoded. If present, the ‘D’ following the peak designation indicates that DTW was used. The next part indicates the distance measure used (Cos = cosine or Euc = Euclidean). The final part indicates the dendrogram reconstruction method‘UP’ indicates UPGMA.

**Figure 2.**
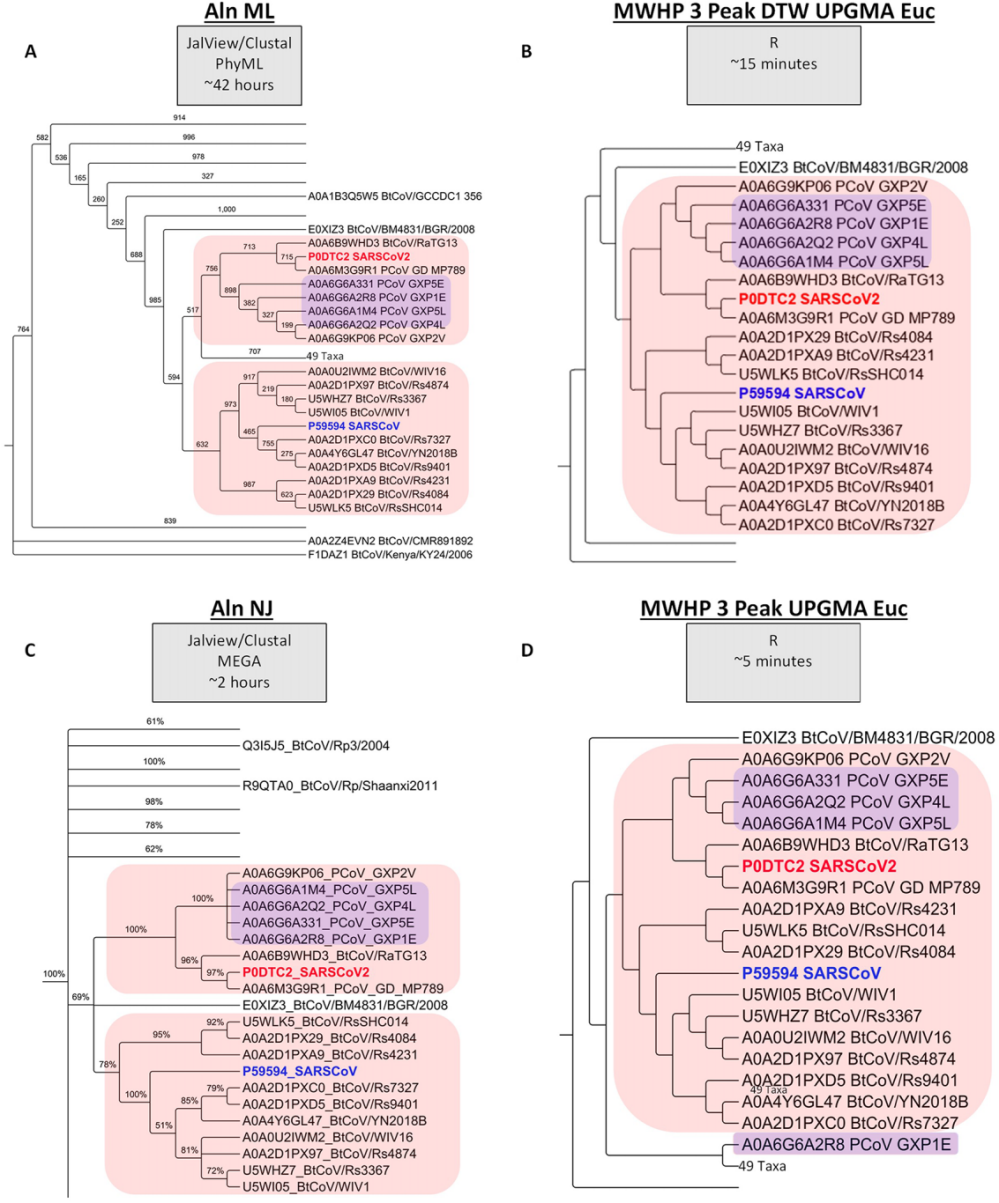
(A) Dendrogram depiction of ML phylogenetic tree produced from a multiple alignment of RBD’s from betacoronaviruses. The values are the number of supporting bootstrap steps out of 1000. (B) Dendrogram of Betacoronaviruses constructed using DTW with Euclidean distance and hierarchical clustering of the herein described waveform representations (three-peaks) of RBD sequences. (C) Dendrogram depiction of NJ phylogenetic tree produced from a multiple alignment of RBD’s from Betacoronaviruses. (D) Dendrogram of Betacoronaviruses constructed using Euclidean distance and hierarchical clustering of the herein described waveform representations (three-peaks) of RBD sequences. The values shown are the supporting percentage of 1000 bootstrap steps. All dendrograms have been cropped for display purposes. The full dendrogram for each of these trees can be found in the Supplementary Materials.

The MW and HP of amino acids were used to encode the RBDs of the betacoronavirus spike proteins. Those encoded sequences were subjected to direct comparisons using Euclidean and cosine distance and also DTW comparison using Euclidean distance. The resulting distance matrices were then subjected to hierarchical clustering analysis using both NJ and UPGMA methodologies and compared to the ML and NJ trees produced using a multiple alignment of the same primary sequences. As can be observed in Fig.  1, several variations of our analysis were performed with single-peak and three-peak amino acid waveforms. All dendrograms produced using hierarchical clustering with either direct Euclidean or cosine comparisons or DTW Euclidean comparison resulted in all coronavirus RBDs known to bind ACE2, clustered in a single well-formed group (Fig.  2B and D and Supplementary Figs S3–S14). The non-DTW waveform-based analyses (Fig.  2D and Supplementary Figs S7–S14) resulted in a single pangolin RBD sequence (GXP1E) outside of that cluster.

**Figure 3.**
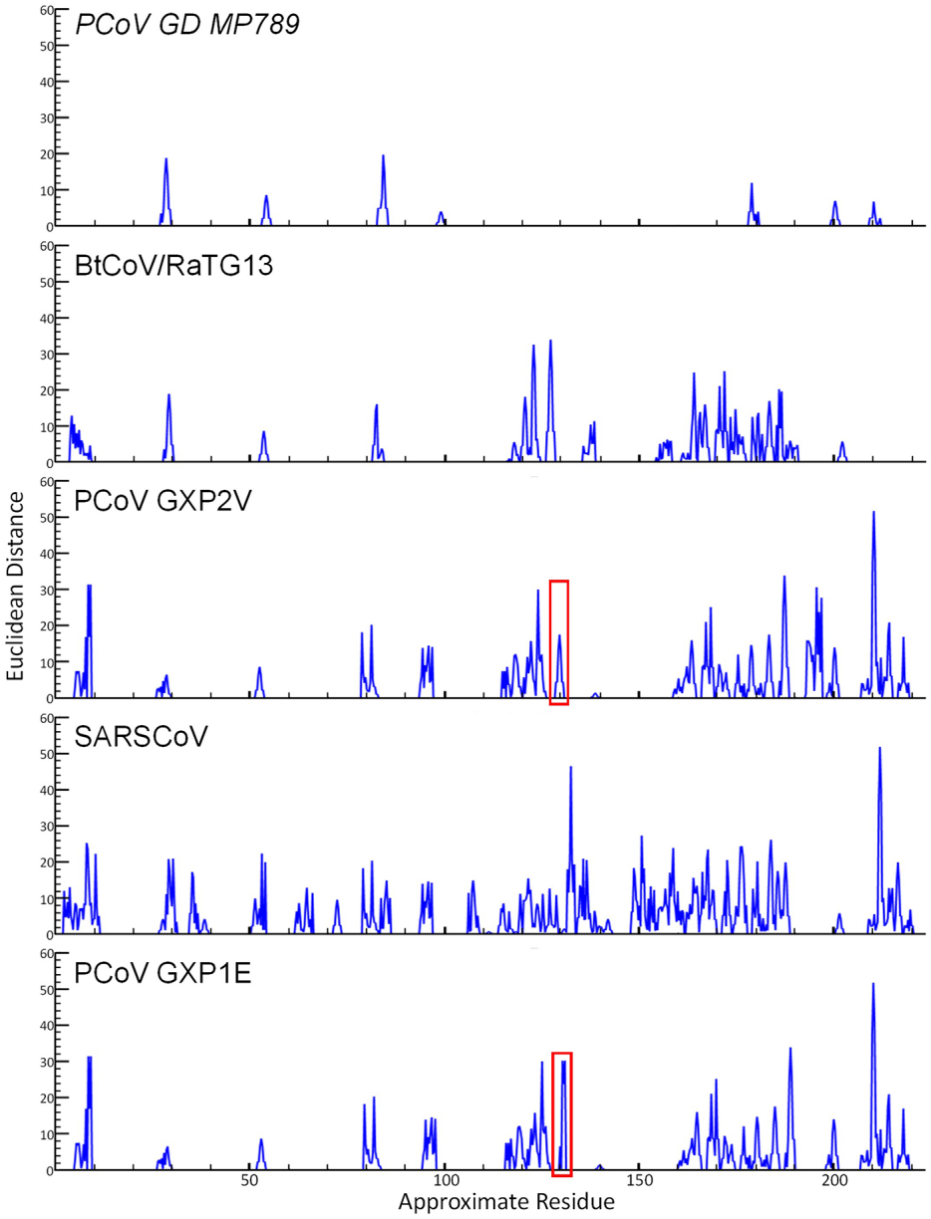
A DTW comparison of the waveforms of each of the indicated coronavirus RBD’s to that of SARS-CoV-2. The comparisons are ordered from top to bottom respective of how close they cluster with SARS-CoV-2 in the non-DTW dendrogram depicted in Fig.  2D. Despite minimal divergence between GXP2V and GXP1E as indicated by Euclidean distance here and among the four critical ACE2 contact residues in Fig.  4, GXP1E does not tightly cluster with known ACE2-binding RBD’s in Fig.  2D. We attribute this to the single peak marked with red boxes, where GXP1E is more divergent from SARS-CoV-2 than GXP2V is. That peak corresponds to two amino acid deletion in GXP1E in Fig.  4. Of further interest is that despite GXP1E being clearly less divergent from SARS-CoV-2 than SARS-CoV is here, SARS-CoV clusters with SARS-CoV-2 in Fig.  2D, whereas GXP1E does not, potentially indicating the ability of strict Euclidean distance, without DTW, to resolve key differences with respect to ACE2 binding. Residue positions on the *X*-axis should be considered approximate due to the variable nature of dynamic time warping with regard to the stretch/compression of the *X*-axis as related to the particular waveform pair in each comparison.

While alignment-based ML and NJ are both commonly used in the analysis of protein sequences, the congruency between the dendrograms produced using these methods was low as indicated by tanglegram comparison of those dendrograms in Supplementary Fig. S2 and the Robinson–Foulds Metric value of 0.53 (Fig.  1B), despite 77% common nodes among them as shown in Fig.  1A. There is only slightly lower congruency, as measured by common nodes in Fig.  1a, between the widely accepted and utilized ML method and the DTW methods employed herein (75–76% common nodes). The Robinson–Foulds Metric values from the comparisons of alignment-free DTW dendrograms to the alignment-based ML dendrograms are equal to or higher than those for the comparison to the alignment-based NJ dendrograms at 0.53–0.58 (Fig.  1B). Furthermore, manual comparison of the DTW-based dendrograms (Supplementary Figs S3–S6) to the alignment-based NJ dendrograms (Supplementary Fig. S2) reveals that not only are polytomies common in the alignment-based NJ dendrograms and absent in the DTW-based dendrograms but also that the overall congruency of topology is greater in the DTW-based analyses. Therefore, the alignment-based NJ method was considered unsuitable to the analysis of coronavirus spike protein RBDs, an observation that to our knowledge has not been specifically addressed in the literature but is apparent by the lack of NJ methodology in published studies regarding the evolutionary history of the coronavirus spike protein RBD. In an attempt to better resolve the polytomies present in the NJ tree, we removed 28 basal RBDs from the dataset because those sequences were members of clades with low support and multiple polytomies. Re-alignment and NJ tree construction using the truncated dataset resulted in fewer polytomies and a majority of nodes with support; however, the overall topology remained similar to the original NJ tree, with no changes to the topology of the ACE2-binding clade.

DTW and strict Euclidean/cosine distance-based methods employed herein resulted in little ambiguity within and among clusters and were in general agreement, regarding cluster composition, with the alignment-based ML analysis. However, there is a major exception among ACE2-binding RBDs where the alignment-based ML method results in two separate clusters, a finding that has been reported by others as well [17, 55–58]; in contrast, both the DTW and strict Euclidean/cosine distance methods yield a single consolidated ACE2-binding cluster as shown in Fig.  2B and D as well as in Supplementary Figs S3–S14. Common node comparisons for congruency presented in Fig.  1A indicate that the DTW-based methods result in dendrogram topologies that are more similar to the alignment-based ML topology than the alignment-free strict Euclidean distance dendrograms with regard to cluster composition. Despite this, inter-cluster relatedness varies between the methods as indicated by lower topological agreement as revealed using the Robinson–Foulds Metric in Fig.  1B.

The alignment-based NJ tree shows a less cohesive grouping of ACE2-binding RBDs than does our physicochemical clustering method and also exhibits numerous polytomies (Fig.  2C). Additionally, our algorithms are far less computationally intensive than either alignment-based ML or alignment-based NJ analyses as shown in Fig.  2. Briefly, the ML phylogenetic reconstruction took ∼42 h to complete on the PhyML web server, the NJ phylogenetic reconstruction took ∼2 h, the strict Euclidean distance reconstruction took ∼5 min and the DTW-based method took ∼15 min highlighting a significant reduction of processing time for the alignment-free techniques.

Closer examination of congruency as indicated by the proportion of nodes with the exact same taxa (Common Nodes) in Fig.  1A reveals that the DTW-based techniques recovered 75–76% of the node composition of the dendrogram produced by the alignment-based ML method. In contrast, the strict Euclidean distance-based analyses recovered only 69–73% of the node composition. Among the DTW-based analyses, the greatest level of node composition agreement occurred between the single- and three-peak UPGMA analyses (98%). The agreement between alignment-based ML method and the three-peak UPGMA method was 76% (slightly higher than 75% agreement with the single-peak UPGMA analysis; refer to Fig.  2B for the three-peak dendrogram and Supplementary Fig. S5 for the single-peak dendrogram). The full suite of Common Nodes across all pairwise dendrogram comparisons is displayed in Fig.  1A.

In addition to the cluster composition, overall tree topological congruency was evaluated using the Robinson–Foulds Metric. The greatest level of congruency was observed between the single- and three-peak DTW analyses and the alignment-based ML analysis (58%) as shown in Fig.  1B. This is indicative of a moderate level of similarity in the overall topology of the dendrograms. However, the greatest level of consistency from single- to three-peak DTW analyses was with the UPGMA methodology (96%). The full suite of Robinson–Foulds Metrics across all pairwise dendrogram comparisons is shown in Fig.  1B.

**Figure 4.**
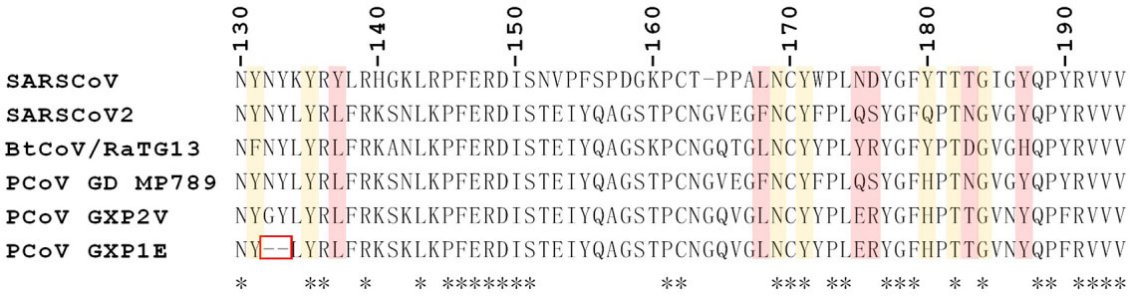
Partial alignment of the RBD for the coronaviruses indicated. Shaded red boxes indicate key residues for binding to the human ACE2 receptor as previously reported, yellow boxes indicate key residues that contact the ACE2 receptor in general [19, 57, 59]. Of note, the Zhang et al. and Lam et al. studies are in disagreement as to whether residue 187 is key in human ACE2 binding or ACE2 binding in general. Wan et al. originally reported that it was not critical in human ACE2 binding [19]. We predict, based on our non-dtw-based bioinformatic approach that GXP1E will not bind to ACE2. It clusters with non-ACE2-binding coronaviruses using strict Euclidean distance techniques (Fig.  2D) and has a two-residue deletion (red box) which corresponds to the peak shown in Fig.  3 where GXP1E (predicted non-ACE2-binding) and GXP2V (ACE2-binding [20]) differ.

Due to multiple polytomies in the tree produced using an alignment-based NJ methodology (Fig.  2C and Supplementary Fig. S2), the topology depicted was deemed unreliable and largely unresolved. Visual comparison of the full suite of dendrograms/trees produced from waveform representations of the amino acid sequences (Supplementary Figs S3–S14) revealed a common theme and a few interesting observations. Regardless of the exact waveform-based methodology, using MW and HP as described herein always resulted in all known ACE2-binding RBDs forming a single consolidated cluster (Fig.  2B and D and Supplementary Figs S3–S14) in contrast to the two separate groups resulting from alignment-based ML methodology (Fig.2A and Supplementary Figs S2–S14). All but one pangolin RBD (GXP1E) clustered within the ACE2-binding group with all non-DTW waveform methodologies (Fig.  2D and Supplementary Figs S7–S14). That finding was further explored by performing a DTW comparison of the waveform representations of the SARS-CoV-2 RBDs on a one-to-one basis as can be seen in Fig.  3. That visual comparison revealed that GXP2V which has been confirmed to bind ACE2 [20] shows more similarity than GXP1E to SARS-CoV-2 in only a single location (∼140 in the waveform comparison). That single location corresponds to residues 132–133 in the amino acid alignment where GXP1E has a two-residue deletion (Fig.  4). That deletion is only four residues away from a key residue in binding of human ACE2 [59] and therefore may result in a conformational change resulting in the loss of ACE2 binding. With the exception of GXP2V and GXP1E, all of the sequences shown in Fig.  3 are identical at positions 132–133 of the primary sequence alignment. However, unlike GXP1E, GXP2V does not have a deletion at those positions. Instead, GXP2V vs. the other sequences known to bind ACE2, exhibits a glycine to asparagine substitution at position 132 and is identical to the other sequences at position 133 (Fig.3).

## DISCUSSION

The death toll from SARS-CoV-2 has been growing steadily since the beginning of the COVID-19 pandemic in early 2020 [60]. Recently, there have been calls for methods that can cost-effectively and quickly predict the potential for any newly discovered coronavirus to make the zoonotic leap to the human population [16]. The ability to bind the ACE2 receptor is key to that transition particularly within SARS-like betacoronaviruses [18, 19]. Alignment-based phylogenetic analyses of the Spike protein RBDs results in two separate clades of ACE2-binding betacoronaviruses as has been found by others [17, 55–58] and is confirmed here (Fig.  2A and Supplementary Figs S2–S14). The new approach described herein provides an avenue to objectively resolve ACE2-binding betacoronaviruses as a single unified cluster using a waveform analysis of the RBD. That single cluster includes SARS-CoV, SARS-CoV-2, 11 ACE2-binding bat coronaviruses, and all of the pangolin RBDs examined using DTW methodology and all but one pangolin coronavirus RBDs (GXP1E) with direct Euclidean or cosine distance measurement.

Four of the pangolin coronavirus RBDs used in our analyses have not been investigated with regards to ACE2 binding. However, two pangolin coronaviruses that have been previously investigated and confirmed to bind ACE2 (GXP2V and MP789) are found within our unified cluster [20, 21] raising the possibility that the unconfirmed ACE2-binding ability of some or all of the other four will ultimately be confirmed. The four yet to be investigated pangolin RBDs are found within the ACE2-binding cluster when DTW analyses are performed (Fig.  2B and Supplementary Figs S3–S6). The placement of GXP1E outside of the ACE2-binding clade, when using non-DTW waveform-based methodology, appears to have been caused by a two-residue deletion that is four residues upstream of a known ACE2 contact residue [59], as shown in Fig.  4. This is perhaps indicative of a consequential conformational change facilitated by those deletions. Additionally, deletions of two residues further upstream have previously been associated with a lack of ACE2 binding [59]. The two-residue deletion shown for GXP1E in Fig.  4 is part of a quasi-repetitive region consisting of the sequence N, Y/F, N/G, and Y, presenting the possibility that due to the repeat structure of this region, the alignment algorithm may have artificially shifted the deletions shown for GXP1E by two residues thus excluding those deletions from the deletion region common to non-ACE2-binding RBDs reported by Zhang et al. [59]. For these reasons, we conclude that GXP1E may not bind to ACE2 and that our non-DTW method was able to predict this with no manual examination of the RBD despite the high level of similarity between GXP1E and GXP2V, a confirmed ACE2-binding betacoronavirus RBD that also clusters with other ACE2-binding betacoronavirus RBDs in our analyses. While this reveals the power of non-DTW waveform-based methods to discriminate between ACE2-binding and non-ACE2-binding RBDs, it should be applied cautiously since without using DTW, any deletion may have an amplified effect on the distance comparisons and may in some cases shift the placement of a single taxon artificially. Furthermore, the non-DTW methodology with regards to physicochemical clustering can only be reliable when intra-cluster indels are rare. In further support of our proposal that GXP1E might not bind to ACE2 despite clustering near or within the ACE2-binding cluster in our waveform-based analyses, all of the RBDs found by Zhang et al. which have deletions in this upstream region and do not bind ACE2, are part of a 49-member cluster that we found to be distinct from the ACE2-binding cluster in all waveform-based analyses (both DTW and non-DTW). Additionally, those 49 taxa were situated between the two clusters of ACE2 binding RBDs in the alignment-based ML analysis (Fig.  2A) yet were not in any of our waveform-based analyses, thus illuminating the ability of our methods to properly cluster ACE2-binding RBDs based on functional relatedness. Our results demonstrate that waveform-based methods, especially DTW-based methods including the ones presented herein, can reliably identify newly sequenced ACE2-binding RBDs with no laborious manual examination of the amino acid sequence or error-prone structural prediction.

While it is the most pronounced benefit, the ability to identify newly sequenced ACE2-binding RBDs is not the only value in our waveform-based approach. Some studies have focused on the divergent origin of SARS-CoV-2 coupled with recombination [55, 61]. Other studies have presented the possibility that while the virus might have arisen via descent, the ability to bind to the ACE2 receptor might alternatively have arisen from a convergent evolutionary process [57]. The genesis of an ACE2-binding RBD and whether or not SARS-CoV-2 emerged due to recombination or convergent evolution are independent questions. The current RBD of SARS-CoV-2 may not have evolved in the progenitor lineage of SARS-CoV-2 but instead have been acquired via recombination. However, that RBD, wherever it came from previously, evolved to the point of ACE2-binding. It is that evolutionary process which our waveform-based methods most notably provide insight into. Despite this, if the alignment-based ML topology shown in Fig.  2A and Supplementary Figs S2–S14 and put forth previously by others [17, 55–58] does not accurately reflect the true evolutionary history of the RBDs and instead the topology revealed by our waveform-based technique (Fig.  2B) is more accurate, then a single clade of ACE2-binding RBDs evolved from their common ancestor and the greatest zoonotic concern is the further expansion of that clade of ACE2-binding RBDs through recombination. However, if the relationships depicted in the ML dendrogram are accurate with regards to the true evolutionary history of the RBDs and the waveform-based dendrograms are merely constructs of convergent structural conformations that deviate from the true evolutionary history of the groups, then a repetitive functional convergent process is of greatest concern. The low level of support for the two separate ACE2-binding clades in the alignment-based ML dendrogram in Fig.  2A (59.4% and 51.7%) draws into question the veracity of the two-clade topology and is therefore circumstantially supportive of the topology derived using our waveform-based method (Fig.  2B) as an indicator of the true evolutionary history of the ACE2-binding betacoronavirus RBDs. Additionally, it is important to consider that convergence and recombination may act in combination and therefore mutual exclusivity should not be assumed and is actually refuted by previous evidence for convergence in the incongruency of dendrograms produced from only the synonymous sites of the RBDs versus the entire genome [57] and also in later topological congruency analyses [62]. Such incongruency among phylogenetic topologies has been reported to oftentimes indicate convergent molecular evolution and appears to be much more common than has been historically accepted [63]. For that reason, it is premature to exclude convergent evolution as the dominant mode of evolution with regard to the acquisition of the ability to bind to ACE2 by betacoronavirus RBDs, especially considering our consolidation of the two groups of ACE2-binding RBDs using physicochemically derived waveforms.

Another way to approach the question of convergent evolution is by asking if there is excessive conservation of function given the observed level of phylogenetic diversification. Such functional conservation can be maintained semi-independent of primary sequence due to overlap in the physicochemical profiles of amino acids. Lolkema and Slotboom noted this physicochemical conservation and therefore made their previously mentioned statement to the effect that the 3D structure of proteins and thus their function is very tolerant of changes in their primary sequence [27]. Herein, our method exploits that principle and in an objective manner, independent of structural modeling allows for the evaluation of physicochemical clustering, which is validated by known functional classification of ACE2-binding RBDs [61] as shown in Fig.  2B and D. Additionally, not only is this an objective method but it is also based on both the content and context of the amino acid sequence, thus preserving the evolutionary assumption rather than creating a spurious metric based merely on shared characteristics. Therefore, conclusions made using this technique should not be considered strictly function-based. Instead, in the case of betacoronavirus RBDs shown in Fig.  2A, B, and D and in totality in Supplementary Figs S2–S14, our method can be used to resolve phylogenetic topologies with weak support using standard methodology such as those leading to the separation of the ACE2-binding RBDs into two clades (Fig.  2A) and may provide more insight into relatedness than standard techniques like alignment-based ML alone. Such complimentary use of standard alignment-based and waveform-based methodologies might be desirable considering that in the absence of the waveform equivalent of multiple sequence alignment, bootstrapping to provide indications of branch support is not possible. Note that the DTW distance matrices are constructed via pairwise comparisons. A valuable future extension to our method would be the development of waveform-guided amino acid alignment similar to current amino acid-guided nucleotide alignment [64]. This may allow for bootstrapping on an amino acid alignment guided by structural conservation.

The true evolutionary history of the ACE2-binding betacoronavirus RBDs may have experienced convergent evolution, recombination or both. However, the most pressing question is whether or not a newly emerged betacoronavirus has the ability to bind to ACE2. Addressing this brings us closer to targeting the betacoronaviruses with the propensity to evolve to bind to human ACE2. The fact that our waveform-based approach results in a single cluster of ACE2-binding betacoronavirus RBDs when a large number of betacoronavirus RBDs were analyzed demonstrates the utility of the method in the identification of potential betacoronavirus zoonoses before they become an actuality in the human population. This is emphasized by the fact that prior to the identification of SARS-CoV-2, it would not have been assumed that a new RBD sequence belonging to the clade that we now know contains SARS-CoV-2 (Fig.  2A) would be a threat to humans on the same scale as SARS-CoV? Developing new approaches to robustly address this or related questions is important and our proposed approach demonstrates a way forward in this direction. Considering the uncertainty surrounding whether SARS-CoV-2 evolved the ability to infect and persist in humans while in a progenitor host, an intermediate host or within a subset of the human population [65], it is imperative that surveillance techniques be employed that can efficiently identify coronaviruses with the potential to experience zoonoses [16]. Our technique requires only amino acid sequence data from the RBD and therefore could easily be used in a metagenomic-based surveillance program. Such surveillance would involve not only monitoring of animal populations such as bats and pangolins but also general surveillance of coronavirus circulation in the human population. Samples for that surveillance could be obtained from numerous sources including clinical samples and waste receptacles in public restrooms. The identification of a new waveform that clusters with the ACE2-binding RBDs would trigger a wider-scale sequencing and clinical monitoring effort aimed at full characterization of the new variant. This could give the scientific community valuable time in the response to any newly emerged betacoronavirus that could potentially have a similar or even higher death rate than either SARS-CoV or SARS-CoV-2. Furthermore, we suggest an expansion of efforts to identify coronaviruses in diverse mammals. It is entirely possible that there exist multiple reservoirs of ACE2-binding betacoronaviruses in animal populations far more diverse than humans, bats, and pangolins. Therefore, worldwide monitoring of diverse animal populations is currently warranted. 

## Supplementary data

Supplementary data is available at *EMPH* online.

**Conflict of interest:** None declared.

## Supplementary Material

eoab032_Supplementary_Data
