# Supplementary material for: A novel predictor of ACE2-binding ability among betacoronaviruses
Source: Evol Med Public Health. 2021 Oct 13;9(1):360–73. doi: 10.1093/emph/eoab032 (PMC8634463; doi:10.1093/emph/eoab032)
Supplement: eoab032_Supplementary_Data [file eoab032_supplementary_data.zip › Source Code.pdf]

````
---
title: "MWHP Conversion and DTW Hierarchical Clustering"
author: "Jamie Dixson"
date: "4/10/2021"
output:
  pdf_document: default
  html_document: default
---

```{r setup, include=FALSE}
knitr::opts_chunk$set(echo = TRUE)
```
````

Molecular Weight and Hydrophobicity Clustering using DTW

Waveform Conversion Script

The following script converts the amino acid sequences in a FASTA file to vector form, develops a dtw distance matrix and performs hierarchical clustering. The conversion to waveform can be conducted with any vector representing the properties or combined properties of each amino acid. The stock script shown here uses a combination of molecular weight and hydrophobicity. The vector to change if you want to use another property/properties is NoteArray and it should be in the order GALMFWKQESPVICYHRNDT.

````
```{r}
#Set the input Fasta file location (protein sequences only).
InputFile<-"C:/Path to File/ExampleData.fasta"

#Set what part of the FASTA Annotation to use.
#Can be FALSE or TRUE. If FALSE then the entire annotation will be used as
the name. If TRUE then just the name will be used.
UseNamesTF<-FALSE

#Set the output folder.This should be in the form "c:/Folder_Name/"
OutputFolder<-"C:/Path to Output Directory/"

#Set the output name.  This is what you want to call you individual output
files.  Two files will be created.  One will be a newick tree file and the
other will be a pdf showing the tree.
OutputName<-"ExampleOutput"

#This is where you set whether to use single peaks or three peaks the
variable should be set to TRUE or FALSE
threepeaks<-FALSE

#####################Don't Change Anything Below
Here#####################################

#This imports the seqinr and stringr libraries and opens the FASTA file of
protein sequences
library(stringr)
library(seqinr)
library(dtw)
input<-read.fasta(InputFile,as.string = TRUE, seqtype = "AA",seqonly=FALSE)

#This initializes an empty dataframe to hold the vector sequences
waveformsdf<-data.frame("Name","Point",check.names = FALSE)
names(waveformsdf) = gsub('"', "", names(waveformsdf))

#This iterates through all of the sequences in the FASTA file
for (j in 1:length(input)){
````
````

```
  #This gets the information for the current sequence
  if(UseNamesTF==FALSE){
  name<-getAnnot(input[j])#use getAnnot for the full description or getName
for just the name
  name<-substr(name,2,nchar(name))
  }
  else{
  name<-getName(input[j])#use getAnnot for the full description or getName
for just the name
  name<-substr(name,1,nchar(name))
  }

  currentseq<-getSequence(input[j])
  currentseq<-unlist(currentseq,recursive = TRUE, use.names = FALSE)
  lengther<-length(currentseq)

  #If a character other than "ACDEFGHIKLMNPQRSTVWYacdefghiklmnpqrstvwy" is
found then we will replace it with "G".  All    #lower case residues are
converted to upper case.  The currentseq is ready for analysis after this
block of code.
  #library(stringr)
  replacecounter<-0
  for(i in 1:lengther){
  StringtoConvert<-currentseq[i]
  strAllowedChars="ACDEFGHIKLMNPQRSTVWYacdefghiklmnpqrstvwy"
  outstring<-
str_replace_all(StringtoConvert,paste0("[^",strAllowedChars,"]"),"G")
  if (currentseq[i]!=outstring){replacecounter<-replacecounter+1
  outstring<-toupper(outstring)
  currentseq[i]<-outstring}
  else{}
  }

  #This creates the list that will be used to calculate the values in the
vector.The NoteArray values shown represent the   #residue MW divided by
the MW of the most massive AA residue X the hydrophobicity index. The
values are in the order     #GALMFWKQESPVICYHRNDT.
  NoteArray<-c(0.3064, 15.6497, 60.7668, 52.1362, 76.6645, 97, -15.8315, -
6.8806, 5.547, -2.3381, -23.9905, 40.4569, 60.1592, 27.1418, 42.9375, -
22.8297, -11.7423, -17.1576, -33.9918, 7.0585)

  #This initializes the ForeTempoArray and AftTempoArray
  ForeTempoArray<-outer(NoteArray, NoteArray, "+") / 2
  ForeTempoArray<-round(ForeTempoArray,3)
  AftTempoArray<-round(ForeTempoArray,3)

  #This initializes the variable that will hold the converted sequence
  StringConverted<-""

  #This works through the sequence one residue at a time
  for (i in 1:lengther){

    #This sets the note value based on the current residue
    if(toupper(currentseq[i]=="G")){NoteX=1}
    else if(toupper(currentseq[i]=="A")){NoteX=2}
    else if(toupper(currentseq[i]=="L")){NoteX=3}
    else if(toupper(currentseq[i]=="M")){NoteX=4}
    else if(toupper(currentseq[i]=="F")){NoteX=5}
    else if(toupper(currentseq[i]=="W")){NoteX=6}
    else if(toupper(currentseq[i]=="K")){NoteX=7}
    else if(toupper(currentseq[i]=="Q")){NoteX=8}
    else if(toupper(currentseq[i]=="E")){NoteX=9}
    else if(toupper(currentseq[i]=="S")){NoteX=10}
    else if(toupper(currentseq[i]=="P")){NoteX=11}
    else if(toupper(currentseq[i]=="V")){NoteX=12}
```

```
else if(toupper(currentseq[i]=="I")){NoteX=13}
else if(toupper(currentseq[i]=="C")){NoteX=14}
else if(toupper(currentseq[i]=="Y")){NoteX=15}
else if(toupper(currentseq[i]=="H")){NoteX=16}
else if(toupper(currentseq[i]=="R")){NoteX=17}
else if(toupper(currentseq[i]=="N")){NoteX=18}
else if(toupper(currentseq[i]=="D")){NoteX=19}
else if(toupper(currentseq[i]=="T")){NoteX=20}
else{}

#This sets the SearchX1 variable
  SearchX1=NoteX

#This sets the SearchY1 variable
if(i!=1){
    if(toupper(currentseq[i-1]=="G")){SearchY1=1}
  else if(toupper(currentseq[i-1]=="A")){SearchY1=2}
  else if(toupper(currentseq[i-1]=="L")){SearchY1=3}
  else if(toupper(currentseq[i-1]=="M")){SearchY1=4}
  else if(toupper(currentseq[i-1]=="F")){SearchY1=5}
  else if(toupper(currentseq[i-1]=="W")){SearchY1=6}
  else if(toupper(currentseq[i-1]=="K")){SearchY1=7}
  else if(toupper(currentseq[i-1]=="Q")){SearchY1=8}
  else if(toupper(currentseq[i-1]=="E")){SearchY1=9}
  else if(toupper(currentseq[i-1]=="S")){SearchY1=10}
  else if(toupper(currentseq[i-1]=="P")){SearchY1=11}
  else if(toupper(currentseq[i-1]=="V")){SearchY1=12}
  else if(toupper(currentseq[i-1]=="I")){SearchY1=13}
  else if(toupper(currentseq[i-1]=="C")){SearchY1=14}
  else if(toupper(currentseq[i-1]=="Y")){SearchY1=15}
  else if(toupper(currentseq[i-1]=="H")){SearchY1=16}
  else if(toupper(currentseq[i-1]=="R")){SearchY1=17}
  else if(toupper(currentseq[i-1]=="N")){SearchY1=18}
  else if(toupper(currentseq[i-1]=="D")){SearchY1=19}
  else if(toupper(currentseq[i-1]=="T")){SearchY1=20}
  else{}
  }
else{SearchY1=1}

 #This sets the SearchX2 variable
if(i!=lengther){
  SearchX2<-NoteX
}
else{SearchX2=NoteX}

#This sets the SearchY2 variable
if(i!=lengther){
    if(toupper(currentseq[i+1]=="G")){SearchY2=1}
  else if(toupper(currentseq[i+1]=="A")){SearchY2=2}
  else if(toupper(currentseq[i+1]=="L")){SearchY2=3}
  else if(toupper(currentseq[i+1]=="M")){SearchY2=4}
  else if(toupper(currentseq[i+1]=="F")){SearchY2=5}
  else if(toupper(currentseq[i+1]=="W")){SearchY2=6}
  else if(toupper(currentseq[i+1]=="K")){SearchY2=7}
  else if(toupper(currentseq[i+1]=="Q")){SearchY2=8}
  else if(toupper(currentseq[i+1]=="E")){SearchY2=9}
  else if(toupper(currentseq[i+1]=="S")){SearchY2=10}
  else if(toupper(currentseq[i+1]=="P")){SearchY2=11}
  else if(toupper(currentseq[i+1]=="V")){SearchY2=12}
  else if(toupper(currentseq[i+1]=="I")){SearchY2=13}
  else if(toupper(currentseq[i+1]=="C")){SearchY2=14}
  else if(toupper(currentseq[i+1]=="Y")){SearchY2=15}
  else if(toupper(currentseq[i+1]=="H")){SearchY2=16}
  else if(toupper(currentseq[i+1]=="R")){SearchY2=17}
  else if(toupper(currentseq[i+1]=="N")){SearchY2=18}
  else if(toupper(currentseq[i+1]=="D")){SearchY2=19}
```

```
      else if(toupper(currentseq[i+1]=="T")){SearchY2=20}
      else{}
      }
    else{SearchY2=NoteX}

    #This sets the foretempoavg to the current NoteArray(NoteX) value if
the current character is the
    #first character in the sequence.  THIS BLOCK WORKS CORRECTLY
      if(i==1){ForeTempoAvg<-NoteArray[NoteX]}
      else{ForeTempoAvg<-
(ForeTempoArray[SearchX1,SearchY1]+NoteArray[NoteX])/2}

    #This sets the afttempoavg to the current NoteArray(NoteX) value if the
current character is the last
    #character in the sequence
      if(i==lengther){AftTempoAvg<-NoteArray[NoteX]}
      else{AftTempoAvg<-
(AftTempoArray[SearchX2,SearchY2]+NoteArray[NoteX])/2}

      if(threepeaks==TRUE){
      #Each residue gets three peaks.  This creates the first one.
      CalculatedY<-ForeTempoAvg
      CalculatedY<-round(CalculatedY, digits=3)
      StringConverted<-paste(StringConverted,CalculatedY,",")
      }
      else{}


    #Each residue gets three peaks.  This creates the second one.
    CalculatedY<-
(NoteArray[NoteX]+ForeTempoArray[SearchX1,SearchY1]+AftTempoArray[SearchX2,SearchY2])/
2
    CalculatedY<-round(CalculatedY, digits=3)
    StringConverted<-paste(StringConverted,CalculatedY,",")

      if(threepeaks==TRUE){
      #Each residue gets three peaks.  This creates the third one.
      CalculatedY<-AftTempoAvg
      CalculatedY<-round(CalculatedY, digits=3)
      StringConverted<-paste(StringConverted,CalculatedY,",")
      }
      else{}

  }

  #This trims the comma from the end of the string and prints the string.
  StringConverted<-substr(StringConverted,1,nchar(StringConverted)-1)

  #This puts the converted sequences into a dataframe that can be accessed
later.
  waveformsdf[j,1]<-name
  waveformsdf[j,2]<-StringConverted

}


library(splitstackshape)

#This will preserve the list structure to use with dtw
waveformsdfaslist<-waveformsdf
waveformsdfaslist <- data.frame(waveformsdfaslist, row.names = 1)


#Convert the csv values in the waveformsdf to individual cell values
waveformsdf<-cSplit(waveformsdf,"Point",",")
```

```
#Set the first column as the name column
waveformsdf <- data.frame(waveformsdf, row.names = 1)



### Time Series Clustering Script
#The following script creates a distance matrix among 2+ time series based
on a dynamic time warping derived measure of distance.
#This block of code performs DTWdist on the sequences entered above.  This
one works right!

#This turns off warnings
options(warn=-1)

#waveformsdfaslist2 <- split(waveformsdfaslist,
seq(nrow(waveformsdfaslist)))
waveformsdfaslist2 <-
setNames(split(waveformsdfaslist,seq(nrow(waveformsdfaslist))),rownames(waveformsdfaslist)
)
waveformsdfaslist2<-
lapply(waveformsdfaslist2,function(x)as.numeric(trimws(strsplit(x$Point,',\\s*')[[1]]))
)

#This works if seqsr is a simple list of vectors.  The dist.method variable
if set to Cosine is uninformative since it results in distances of mostly
0.  Euclidean is the better metric here because with perfectly aligned time
series, there is still distance in the differences between peaks and
valleys.
distmatfromdtw <- proxy::dist(waveformsdfaslist2, method =
"DTW",dist.method="Euclidean",dist.step="asymmetric", dist.open.end =
TRUE,dist.open.begin=TRUE, upper = TRUE, diag = TRUE)
#print(distmatfromdtw)

#It can be checked like this
#library(TSdist)
#DTWDistance(seqA,seqB)
#paste("The distance between the two time series using dtw in the TSdist
library is:",DTWDistance(seqA,seqB))

#Hierarchical Clustering
library(TreeTools)
library(ape)

#Hierarchical Cluster the data based on average distance
hc<-hclust(distmatfromdtw,method='average')#Average is the same as UPGMA
plot(hc, cex=0.35, hang=-1)

##Write the tree to a file in newick format
class(hc) # must be hclust class
my_tree <- as.phylo(hc)
OutputFileName1<-paste(OutputName,".nwk")
setwd(OutputFolder)
write.tree(phy=my_tree, file=OutputFileName1) # look for the file in your
working directory

##Write the tree to a file in pdf
library(phytools)
OutputFileName2<-paste(OutputName,".pdf")
setwd(OutputFolder)
pdf(file=OutputFileName2,height=10,width=7.5)
plot(my_tree,type="phylogram",cex=0.5)

#This turns warning back on
options(warn=0)
```

````
```{r}
library(ape)
NJTree<-nj(distmatfromdtw)

##Write the tree to a file in newick format
class(hc) # must be hclust class
my_tree2 <- as.phylo(NJTree)
OutputFileNameNJ1<-paste(OutputName,"NJ.nwk")
setwd(OutputFolder)
write.tree(phy=my_tree2, file=OutputFileNameNJ1) # look for the file in
your working directory

##Write the tree to a file in pdf
library(phytools)
OutputFileNameNJ2<-paste(OutputName,"NJ.pdf")
setwd(OutputFolder)
pdf(file=OutputFileNameNJ2,height=10,width=7.5)
plot(NJTree,type="phylogram",cex=0.5)

```
````
