# Supplementary material for: A novel predictor of ACE2-binding ability among betacoronaviruses
Source: Evol Med Public Health. 2021 Oct 13;9(1):360–73. doi: 10.1093/emph/eoab032 (PMC8634463; doi:10.1093/emph/eoab032)

S1: Full list of sequences used in all analyses with UniProt accession numbers.

#	Acc. #	Identifier
1	A0A2D1PXA9	BtCoV/Rs4231
2	U5WLK5	BtCoV/RsSHC014
3	A0A2D1PX29	BtCoV/Rs4084
4	U5WI05	BtCoV/WIV1
5	U5WHZ7	BtCoV/Rs3367
6	A0A2D1PX97	BtCoV/Rs4874
7	A0A0U2IWM2	BtCoV/WIV16
8	A0A2D1PXD5	BtCoV/Rs9401
9	A0A4Y6GL47	BtCoV/YN2018B
10	A0A2D1PXC0	BtCoV/Rs7327
11	P59594	SARSCoV
12	A0A6G9KP06	PCoV GXP2V
13	A0A6G6A331	PCoV GXP5E
14	A0A6G6A2Q2	PCoV GXP4L
15	A0A6G6A1M4	PCoV GXP5L
16	A0A6B9WHD3	BtCoV/RaTG13
17	P0DTC2	SARSCoV2
18	A0A6M3G9R1	PCoV GD MP789
19	A0A6G6A2R8	PCoV GXP1E
20	A0A096XNM6	BtCoV/Longquan140
21	D5HJV8	BtCoV/HKU39
22	D5HJR2	BtCoV/HKU35
23	D5HJZ1	BtCoV/HKU312
24	D5HJQ1	BtCoV/HKU34
25	D5HJS3	BtCoV/HKU36
26	D5HJW9	BtCoV/HKU310
27	Q3LZT7	BtCoV/HKU33
28	D5HK02	BtCoV/HKU313
29	Q3LZX1	SARSrBtCoV/HKU3
30	Q3LZV3	BtCoV/HKU32
31	A0A0U1WHJ8	BtCoV/GX2013
32	A0A4Y6GL75	BtCoV/YN2018A
33	A0A2D1PX86	BtCoV/Rs4247
34	D5HJT4	BtCoV/HKU37
35	D5HJU5	BtCoV/HKU38
36	A0A4Y6GL43	BtCoV/SC2018
37	A0A0U1WHI2	BtCoV/HuB2013
38	R9QTH3	BtCoV/Cp/Yunnan2011
39	A0A166ZND9	BtCoV/MLHJC35
40	A0A2D1PX44	BtCoV/Rs4081
41	A0A4Y6GL56	BtCoV/YN2018D
42	A0A4Y6GL82	BtCoV/YN2018C
43	A0A2D1PX05	BtCoV/As6526
44	A0A2D1PX88	BtCoV/Rs4255
45	A0A2D1PX73	BtCoV/Rs4237
46	Q3I5J5	BtCoV/Rp3/2004
47	Q0Q475	BtCoV/279/2005
48	Q0QDX9	BtCoV/Rm1/2004
49	R9QTA0	BtCoV/Rp/Shaanxi2011
50	A0A0U1WJY8	BtCoV/YN2013
51	A0A1W5YKT9	BtCoV/Anlong103
52	A0A173G2Q3	BtCoV/B1521
53	A0A0U1UYX4	BtCoV/JL2012
54	A0A221ZS09	BtCoV/16BO133
55	A0A166ZL64	BtCoV/JTMC15
56	A0A0U1WHL1	BtCoV/HuB2013
57	A0A1W5YKU9	BtCoV/Jiyuan84
58	A0A0U1WHH0	BtCoV/HeB2013
59	A0A0U1WHK9	BtCoV/HeN2013
60	A0A0U1WHI6	BtCoV/SX2013
61	Q0Q484	BtCoV/273/2005
62	Q0QDZ0	BtCoV/Rf1/2004
63	A0A0K1Z074	BtCoV/YNLF 31C
64	A0A0K1Z054	BtCoV/YNLF 34C
65	A0A2R3SUW7	BtCoV/ZC45
66	A0A2R3SUW9	BtCoV/ZXC21
67	A0A2D1PX37	BtCoV/Rf4092
68	A0A1W6S788	BtCoV/F46
69	U5NJG5	BtCoV/Neoromicia/-PMLPHE1/RSA/2011
70	A0A1W6ASU7	BtCoV/PREDICT/PDF2180
71	A0A0A0Q7F3	MERSCoV
72	A0A2R4KP86	BtMERSrCoV/NL13845
73	A0A2R4KP93	BtMERSrCoV/NL140422
74	A3EXB2	BtCoV/HKU43
75	A3EX94	BtCoV/HKU4/2004
76	A3EXA3	BtCoV/HKU42
77	S4WYD6	BtCoV/HKU4/2004
78	S4WZQ1	BtCoV/HKU4/2004
79	S4WWQ3	BtCoV/HKU4/2004
80	A3EXC1	BtCoV/HKU44
81	A0A1Z1W230	BtCoV/160081
82	S4WYD2	BtCoV/HKU4/2004
83	A0A1Z1W227	BtCoV/160079
84	A0A1Z1W244	BtCoV/161028
85	Q0Q4F2	BtCoV/133/2005
86	A0A0U1WJZ6	BtCoV/GX2012
87	A0A166ZLN4	BtCoV/JPDB144
88	A0A2I4R888	BtCoV/HKU25
89	A0A2I4R8V9	BtCoV/HKU25
90	A0A2I6PIX8	BtCoV//P.khulii/Italy/-20664563/2011
91	S4X422	BtCoV/HKU5/2004
92	A3EXE8	BtCoV/HKU53
93	A3EXF7	BtCoV/HKU55
94	A3EXD9	BtCoV/HKU52
95	A0A5H2WTJ3	BtCoV/VsCoV1
96	Q0Q4F7	BtCoV/A434/2005
97	A0A0U1WHM0	BtCoV/GD2013
98	S4WWS3	BtCoV/HKU5/2004
99	A3EXD0	BtCoV/HKU5/2004
100	A0A1B3Q5W5	BtCoV/GCCDC1 356
101	A3EXJ0	BtCoV/HKU94
102	A3EXH4	BtCoV/HKU92
103	E0ZN44	BtCoV/HKU952
104	E0ZN60	BtCoV/HKU9102
105	E0ZN52	BtCoV/HKU9101
106	E0ZN36	BtCoV/HKU951
107	A3EXI2	BtCoV/HKU93
108	A0A5B9Y142	BtCoV/BtCoV92
109	A0A4Y6GL90	BtCoV/GX2018
110	A0A2P1M5J5	BtCoV/HKU9
111	A3EXG6	BtCoV/HKU9
112	A0A2Z4EVM5	BtCoV/CMR66
113	F1DAZ9	BtCoV/Kenya/KY06/2006
114	A0A2Z4EVN5	BtCoV/CMR900
115	A0A2Z4EVK1	BtCoV/CMR704P12
116	A0A2Z4EVR7	BtCoV/CMR705P13
117	A0A2Z4EVN2	BtCoV/CMR891892
118	F1DAZ1	BtCoV/Kenya/KY24/2006
119	E0XIZ3	BtCoV/BM4831/BGR/2008

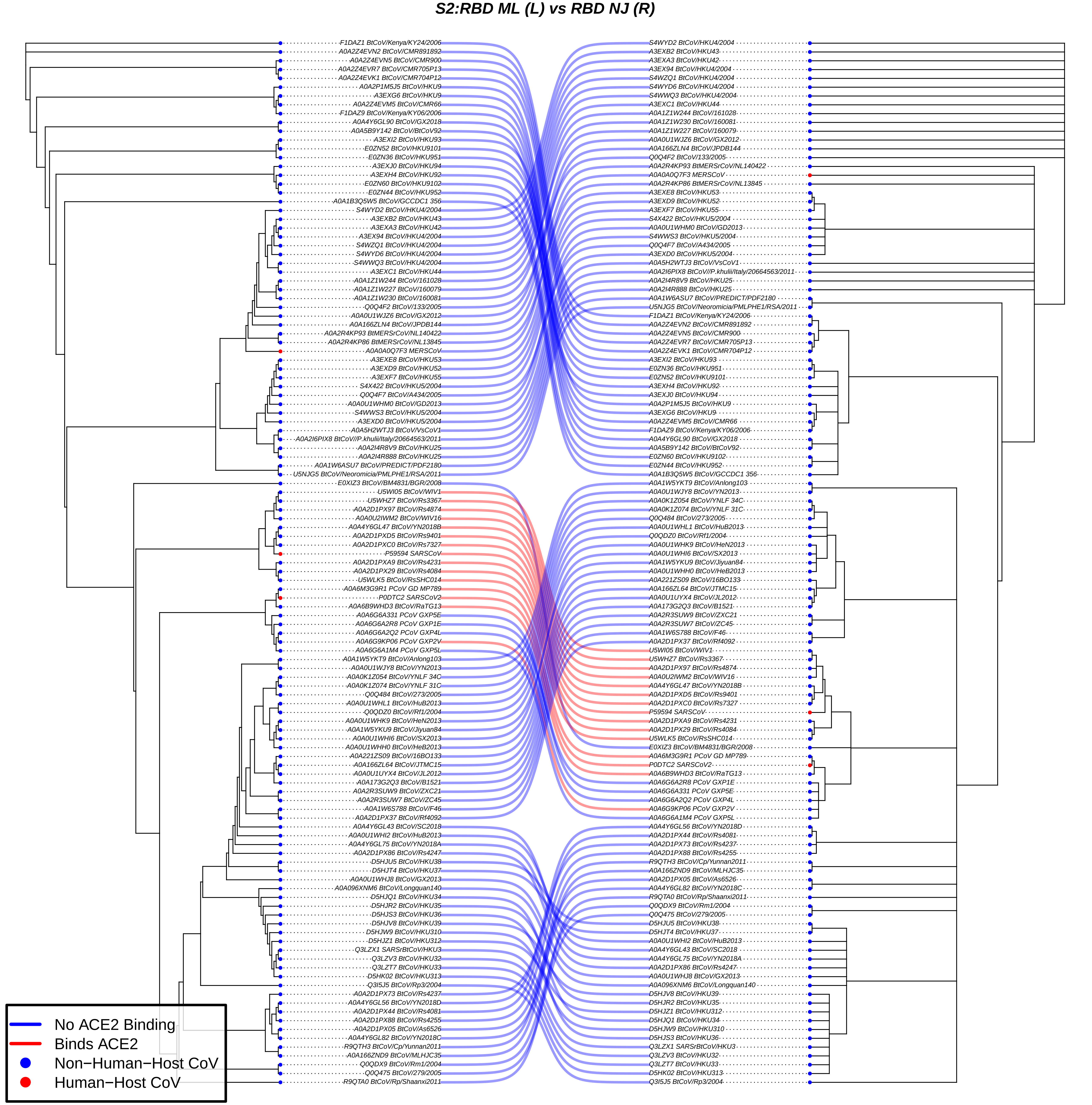
S2:RBD ML (L) vs RBD NJ (R)
F1DAZ1 BtCoV/Kenya/KY24/2006
A0A2Z4EVN2 BtCoV/CMR891892
A0A2Z4EVN5 BtCoV/CMR900
A0A2Z4EVR7 BtCoV/CMR705P13
A0A2Z4EVK1 BtCoV/CMR704P12
A0A2P1M5J5 BtCoV/HKU9
A3EXG6 BtCoV/HKU9
A0A2Z4EVM5 BtCoV/CMR66
F1DAZ9 BtCoV/Kenya/KY06/2006
A0A4Y6GL90 BtCoV/GX2018
A0A5B9Y142 BtCoV/BtCoV92
A3EXI2 BtCoV/HKU93
E0ZN52 BtCoV/HKU9101
E0ZN36 BtCoV/HKU951
A3EXJ0 BtCoV/HKU94
A3EXH4 BtCoV/HKU92
E0ZN60 BtCoV/HKU9102
E0ZN44 BtCoV/HKU952
A0A1B3Q5W5 BtCoV/GCCDC1 356
S4WYD2 BtCoV/HKU4/2004
A3EXB2 BtCoV/HKU43
A3EXA3 BtCoV/HKU42
A3EX94 BtCoV/HKU4/2004
S4WZQ1 BtCoV/HKU4/2004
S4WYD6 BtCoV/HKU4/2004
S4WWQ3 BtCoV/HKU4/2004
A3EXC1 BtCoV/HKU44
A0A1Z1W244 BtCoV/161028
A0A1Z1W227 BtCoV/160079
A0A1Z1W230 BtCoV/160081
Q0Q4F2 BtCoV/133/2005
A0A0U1WJZ6 BtCoV/GX2012
A0A166ZLN4 BtCoV/JPDB144
A0A2R4KP93 BtMERSrCoV/NL140422
A0A2R4KP86 BtMERSrCoV/NL13845
A0A0A0Q7F3 MERSCoV
A3EXE8 BtCoV/HKU53
A3EXD9 BtCoV/HKU52
A3EXF7 BtCoV/HKU55
S4X422 BtCoV/HKU5/2004
Q0Q4F7 BtCoV/A434/2005
A0A0U1WHM0 BtCoV/GD2013
S4WWS3 BtCoV/HKU5/2004
A3EXD0 BtCoV/HKU5/2004
A0A5H2WTJ3 BtCoV/VsCoV1
A0A2I6PIX8 BtCoV//P.khulii/Italy/20664563/2011
A0A2I4R8V9 BtCoV/HKU25
A0A2I4R888 BtCoV/HKU25
A0A1W6ASU7 BtCoV/PREDICT/PDF2180
U5NJG5 BtCoV/Neoromicia/PMLPHE1/RSA/2011
E0XIZ3 BtCoV/BM4831/BGR/2008
U5WI05 BtCoV/WIV1
U5WHZ7 BtCoV/Rs3367
A0A2D1PX97 BtCoV/Rs4874
A0A0U2IWM2 BtCoV/WIV16
A0A4Y6GL47 BtCoV/YN2018B
A0A2D1PXD5 BtCoV/Rs9401
A0A2D1PXC0 BtCoV/Rs7327
P59594 SARSCoV
A0A2D1PXA9 BtCoV/Rs4231
A0A2D1PX29 BtCoV/Rs4084
U5WLK5 BtCoV/RsSHC014
A0A6M3G9R1 PCoV GD MP789
P0DTC2 SARSCoV2
A0A6B9WHD3 BtCoV/RaTG13
A0A6G6A331 PCoV GXP5E
A0A6G6A2R8 PCoV GXP1E
A0A6G6A2Q2 PCoV GXP4L
A0A6G9KP06 PCoV GXP2V
A0A6G6A1M4 PCoV GXP5L
A0A1W5YKT9 BtCoV/Anlong103
A0A0U1WJY8 BtCoV/YN2013
A0A0K1Z054 BtCoV/YNLF 34C
A0A0K1Z074 BtCoV/YNLF 31C
Q0Q484 BtCoV/273/2005
A0A0U1WHL1 BtCoV/HuB2013
Q0QDZ0 BtCoV/Rf1/2004
A0A0U1WHK9 BtCoV/HeN2013
A0A1W5YKU9 BtCoV/Jiyuan84
A0A0U1WHI6 BtCoV/SX2013
A0A0U1WHH0 BtCoV/HeB2013
A0A221ZS09 BtCoV/16BO133
A0A166ZL64 BtCoV/JTMC15
A0A0U1UYX4 BtCoV/JL2012
A0A173G2Q3 BtCoV/B1521
A0A2R3SUW9 BtCoV/ZXC21
A0A2R3SUW7 BtCoV/ZC45
A0A1W6S788 BtCoV/F46
A0A2D1PX37 BtCoV/Rf4092
A0A4Y6GL43 BtCoV/SC2018
A0A0U1WHI2 BtCoV/HuB2013
A0A4Y6GL75 BtCoV/YN2018A
A0A2D1PX86 BtCoV/Rs4247
D5HJU5 BtCoV/HKU38
D5HJT4 BtCoV/HKU37
A0A0U1WHJ8 BtCoV/GX2013
A0A096XNM6 BtCoV/Longquan140
D5HJQ1 BtCoV/HKU34
D5HJR2 BtCoV/HKU35
D5HJS3 BtCoV/HKU36
D5HJV8 BtCoV/HKU39
D5HJW9 BtCoV/HKU310
D5HJZ1 BtCoV/HKU312
Q3LZX1 SARSrBtCoV/HKU3
Q3LZV3 BtCoV/HKU32
Q3LZT7 BtCoV/HKU33
D5HK02 BtCoV/HKU313
Q3I5J5 BtCoV/Rp3/2004
A0A2D1PX73 BtCoV/Rs4237
A0A4Y6GL56 BtCoV/YN2018D
A0A2D1PX44 BtCoV/Rs4081
A0A2D1PX88 BtCoV/Rs4255
A0A2D1PX05 BtCoV/As6526
A0A4Y6GL82 BtCoV/YN2018C
R9QTH3 BtCoV/Cp/Yunnan2011
A0A166ZND9 BtCoV/MLHJC35
Q0QDX9 BtCoV/Rm1/2004
Q0Q475 BtCoV/279/2005
R9QTA0 BtCoV/Rp/Shaanxi2011
S4WYD2 BtCoV/HKU4/2004
A3EXB2 BtCoV/HKU43
A3EXA3 BtCoV/HKU42
A3EX94 BtCoV/HKU4/2004
S4WZQ1 BtCoV/HKU4/2004
S4WYD6 BtCoV/HKU4/2004
S4WWQ3 BtCoV/HKU4/2004
A3EXC1 BtCoV/HKU44
A0A1Z1W244 BtCoV/161028
A0A1Z1W230 BtCoV/160081
A0A1Z1W227 BtCoV/160079
A0A0U1WJZ6 BtCoV/GX2012
A0A166ZLN4 BtCoV/JPDB144
Q0Q4F2 BtCoV/133/2005
A0A2R4KP93 BtMERSrCoV/NL140422
A0A0A0Q7F3 MERSCoV
A0A2R4KP86 BtMERSrCoV/NL13845
A3EXE8 BtCoV/HKU53
A3EXD9 BtCoV/HKU52
A3EXF7 BtCoV/HKU55
S4X422 BtCoV/HKU5/2004
A0A0U1WHM0 BtCoV/GD2013
S4WWS3 BtCoV/HKU5/2004
Q0Q4F7 BtCoV/A434/2005
A3EXD0 BtCoV/HKU5/2004
A0A5H2WTJ3 BtCoV/VsCoV1
A0A2I6PIX8 BtCoV//P.khulii/Italy/20664563/2011
A0A2I4R8V9 BtCoV/HKU25
A0A2I4R888 BtCoV/HKU25
A0A1W6ASU7 BtCoV/PREDICT/PDF2180
U5NJG5 BtCoV/Neoromicia/PMLPHE1/RSA/2011
F1DAZ1 BtCoV/Kenya/KY24/2006
A0A2Z4EVN2 BtCoV/CMR891892
A0A2Z4EVN5 BtCoV/CMR900
A0A2Z4EVR7 BtCoV/CMR705P13
A0A2Z4EVK1 BtCoV/CMR704P12
A3EXI2 BtCoV/HKU93
E0ZN36 BtCoV/HKU951
E0ZN52 BtCoV/HKU9101
A3EXH4 BtCoV/HKU92
A3EXJ0 BtCoV/HKU94
A0A2P1M5J5 BtCoV/HKU9
A3EXG6 BtCoV/HKU9
A0A2Z4EVM5 BtCoV/CMR66
F1DAZ9 BtCoV/Kenya/KY06/2006
A0A4Y6GL90 BtCoV/GX2018
A0A5B9Y142 BtCoV/BtCoV92
E0ZN60 BtCoV/HKU9102
E0ZN44 BtCoV/HKU952
A0A1B3Q5W5 BtCoV/GCCDC1 356
A0A1W5YKT9 BtCoV/Anlong103
A0A0U1WJY8 BtCoV/YN2013
A0A0K1Z054 BtCoV/YNLF 34C
A0A0K1Z074 BtCoV/YNLF 31C
Q0Q484 BtCoV/273/2005
A0A0U1WHL1 BtCoV/HuB2013
Q0QDZ0 BtCoV/Rf1/2004
A0A0U1WHK9 BtCoV/HeN2013
A0A0U1WHI6 BtCoV/SX2013
A0A1W5YKU9 BtCoV/Jiyuan84
A0A0U1WHH0 BtCoV/HeB2013
A0A221ZS09 BtCoV/16BO133
A0A166ZL64 BtCoV/JTMC15
A0A0U1UYX4 BtCoV/JL2012
A0A173G2Q3 BtCoV/B1521
A0A2R3SUW9 BtCoV/ZXC21
A0A2R3SUW7 BtCoV/ZC45
A0A1W6S788 BtCoV/F46
A0A2D1PX37 BtCoV/Rf4092
U5WI05 BtCoV/WIV1
U5WHZ7 BtCoV/Rs3367
A0A2D1PX97 BtCoV/Rs4874
A0A0U2IWM2 BtCoV/WIV16
A0A4Y6GL47 BtCoV/YN2018B
A0A2D1PXD5 BtCoV/Rs9401
A0A2D1PXC0 BtCoV/Rs7327
P59594 SARSCoV
A0A2D1PXA9 BtCoV/Rs4231
A0A2D1PX29 BtCoV/Rs4084
U5WLK5 BtCoV/RsSHC014
E0XIZ3 BtCoV/BM4831/BGR/2008
A0A6M3G9R1 PCoV GD MP789
P0DTC2 SARSCoV2
A0A6B9WHD3 BtCoV/RaTG13
A0A6G6A2R8 PCoV GXP1E
A0A6G6A331 PCoV GXP5E
A0A6G6A2Q2 PCoV GXP4L
A0A6G9KP06 PCoV GXP2V
A0A6G6A1M4 PCoV GXP5L
A0A4Y6GL56 BtCoV/YN2018D
A0A2D1PX44 BtCoV/Rs4081
A0A2D1PX73 BtCoV/Rs4237
A0A2D1PX88 BtCoV/Rs4255
R9QTH3 BtCoV/Cp/Yunnan2011
A0A166ZND9 BtCoV/MLHJC35
A0A2D1PX05 BtCoV/As6526
A0A4Y6GL82 BtCoV/YN2018C
R9QTA0 BtCoV/Rp/Shaanxi2011
Q0QDX9 BtCoV/Rm1/2004
Q0Q475 BtCoV/279/2005
D5HJU5 BtCoV/HKU38
D5HJT4 BtCoV/HKU37
A0A0U1WHI2 BtCoV/HuB2013
A0A4Y6GL43 BtCoV/SC2018
A0A4Y6GL75 BtCoV/YN2018A
A0A2D1PX86 BtCoV/Rs4247
A0A0U1WHJ8 BtCoV/GX2013
A0A096XNM6 BtCoV/Longquan140
D5HJV8 BtCoV/HKU39
D5HJR2 BtCoV/HKU35
D5HJZ1 BtCoV/HKU312
D5HJQ1 BtCoV/HKU34
D5HJW9 BtCoV/HKU310
D5HJS3 BtCoV/HKU36
Q3LZX1 SARSrBtCoV/HKU3
Q3LZV3 BtCoV/HKU32
Q3LZT7 BtCoV/HKU33
D5HK02 BtCoV/HKU313
Q3I5J5 BtCoV/Rp3/2004
No ACE2 Binding
Binds ACE2
Non−Human−Host CoV
Human−Host CoV

S3:RBD ML (L) vs 3 D Euc UP (R)

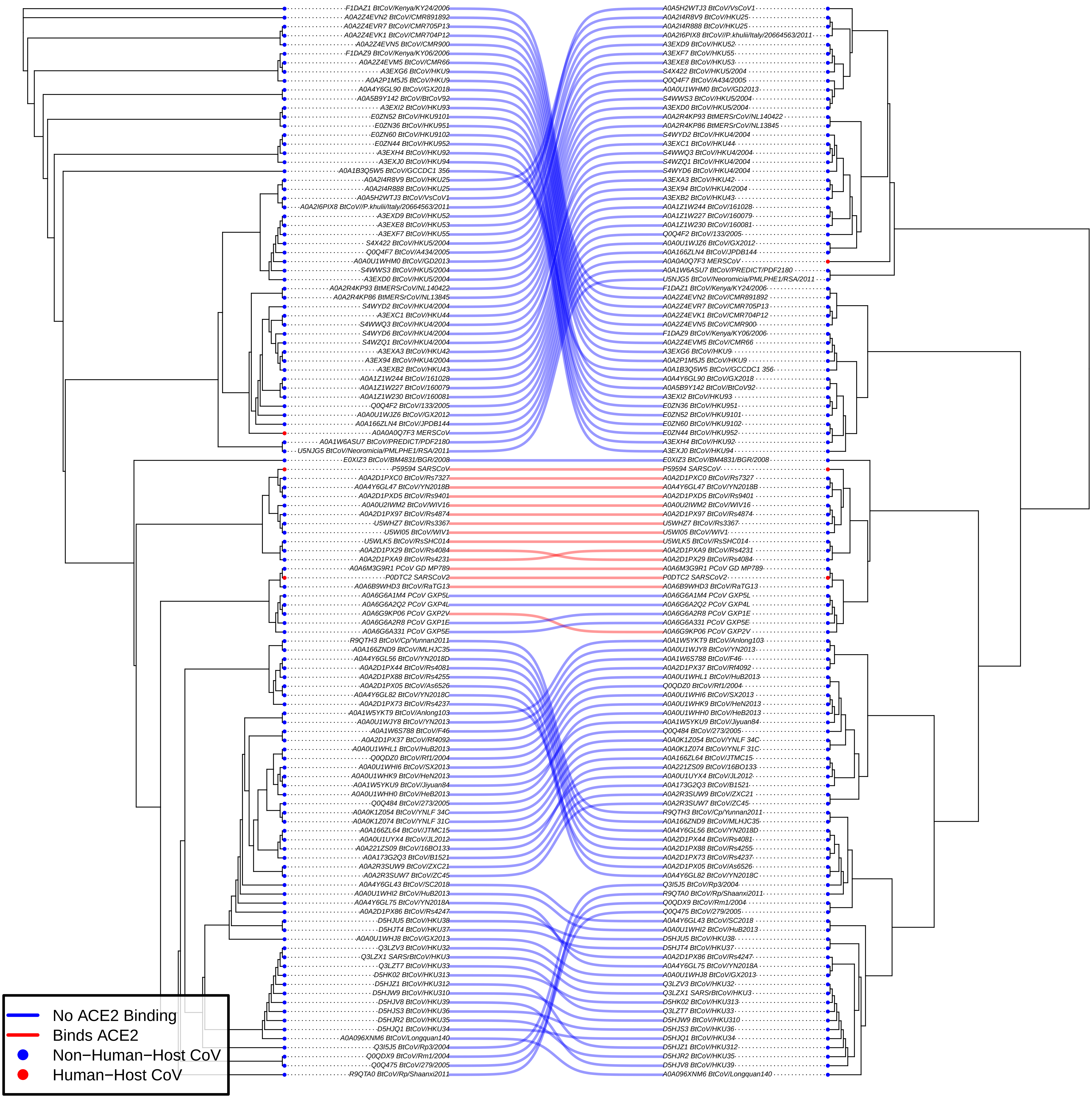

S4:RBD ML (L) vs 3 D Euc NJ (R)

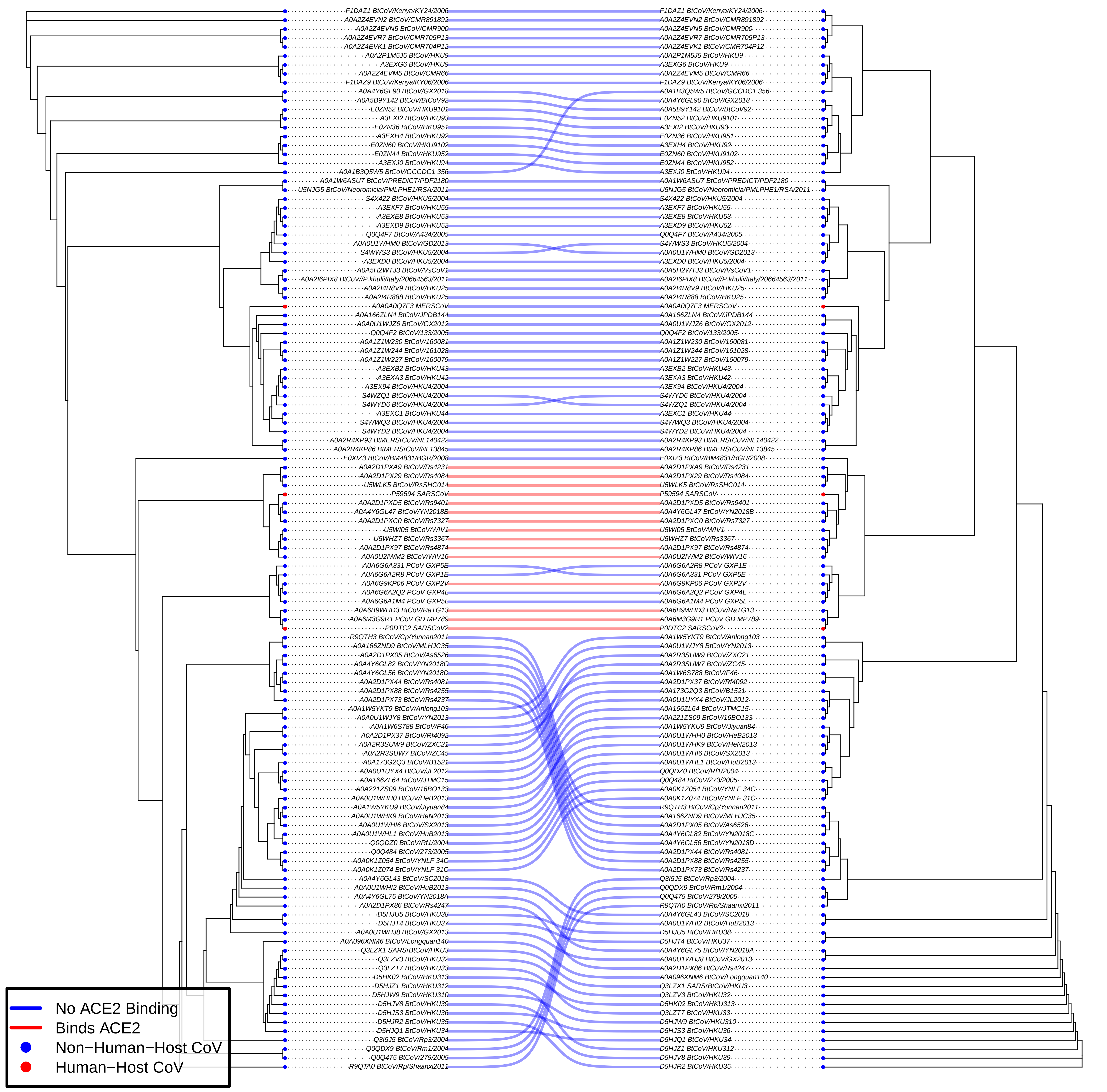

S5:RBD ML (L) vs 1 D Euc UP (R)

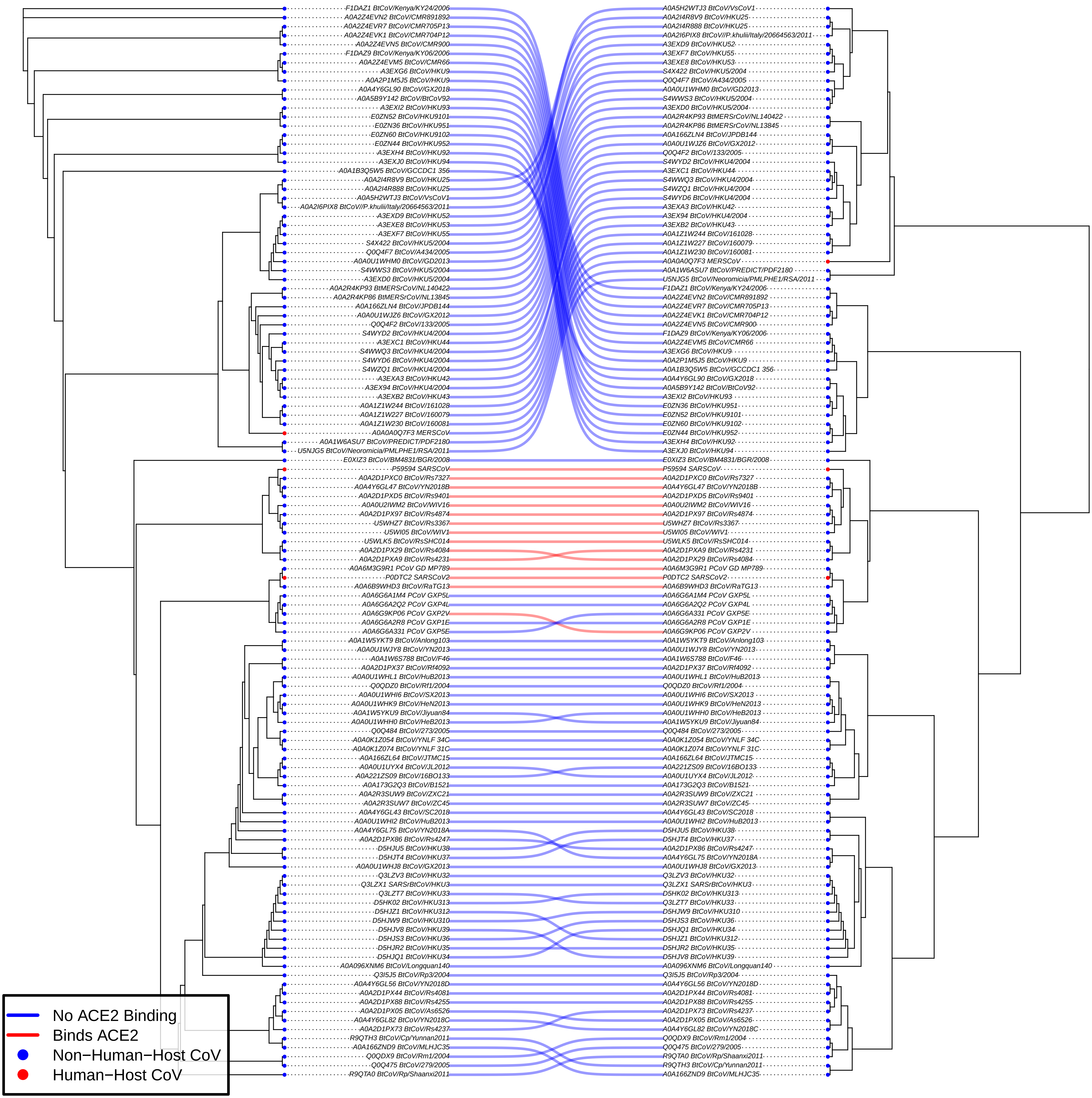

S6:RBD ML (L) vs 1 D Euc NJ (R)

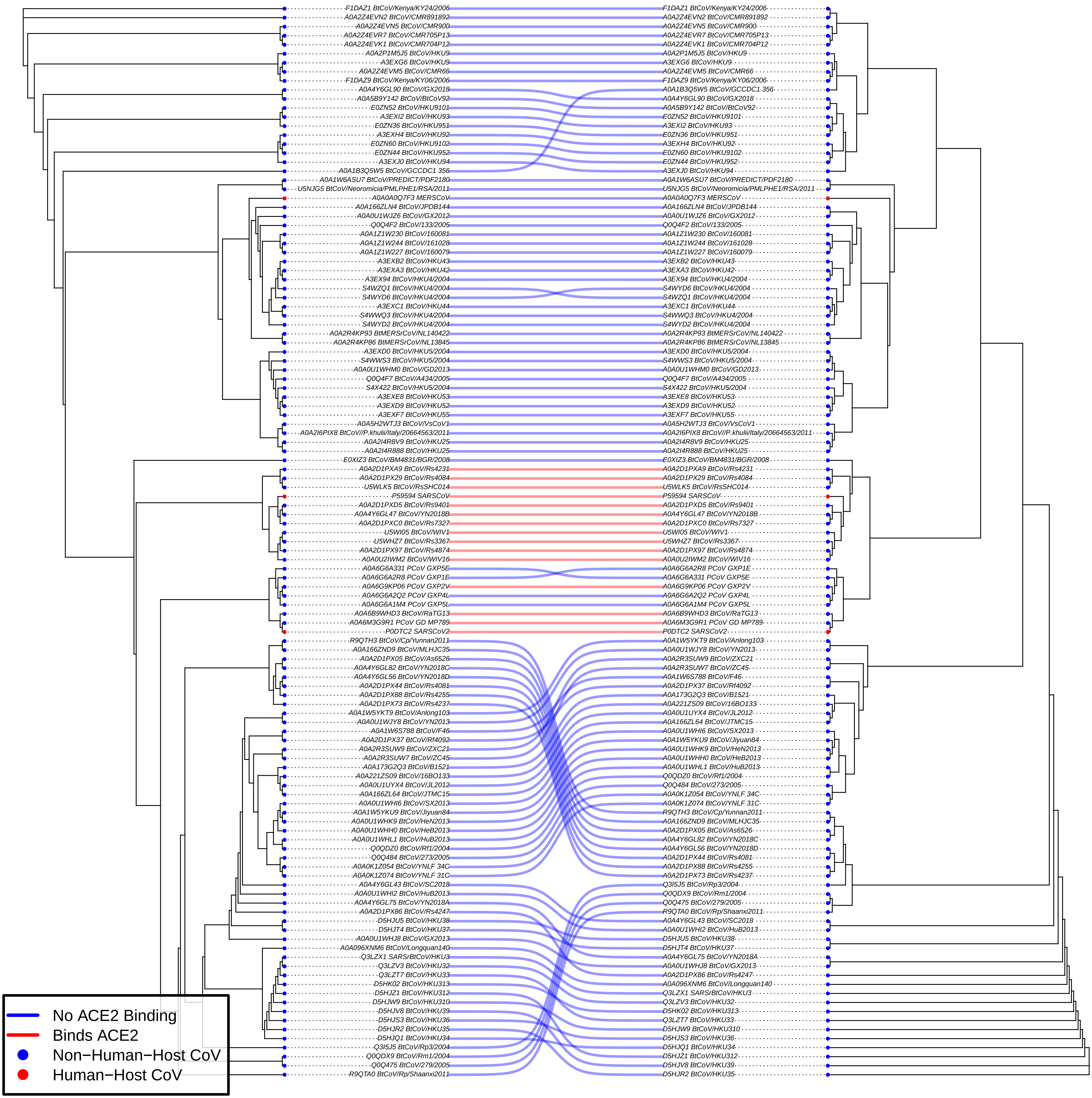

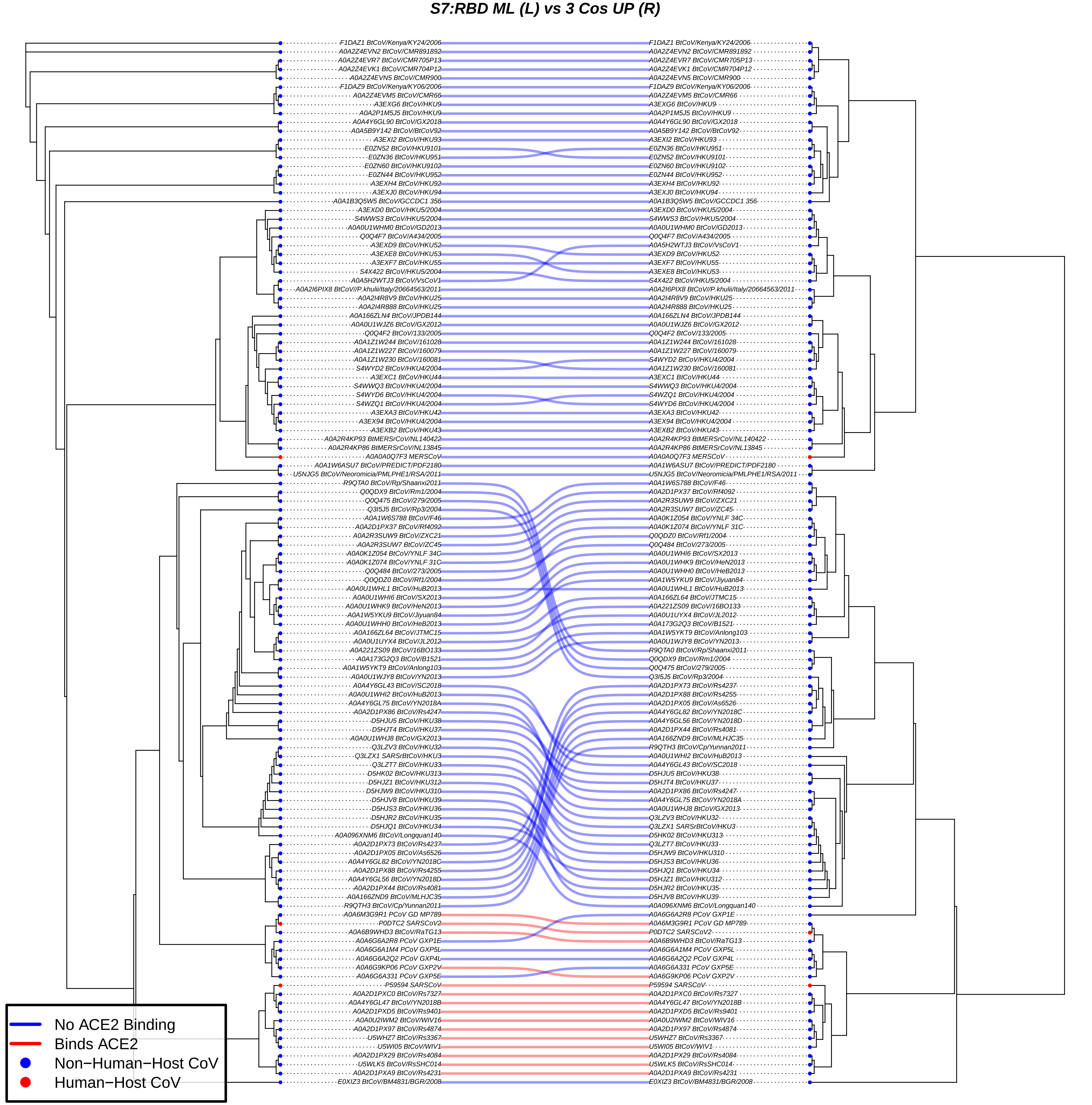
S7:RBD ML (L) vs 3 Cos UP (R)
F1DAZ1 BtCoV/Kenya/KY24/2006
A0A2Z4EVN2 BtCoV/CMR891892
A0A2Z4EVR7 BtCoV/CMR705P13
A0A2Z4EVK1 BtCoV/CMR704P12
A0A2Z4EVN5 BtCoV/CMR900
F1DAZ9 BtCoV/Kenya/KY06/2006
A0A2Z4EVM5 BtCoV/CMR66
A3EXG6 BtCoV/HKU9
A0A2P1M5J5 BtCoV/HKU9
A0A4Y6GL90 BtCoV/GX2018
A0A5B9Y142 BtCoV/BtCoV92
A3EXI2 BtCoV/HKU93
E0ZN52 BtCoV/HKU9101
E0ZN36 BtCoV/HKU951
E0ZN60 BtCoV/HKU9102
E0ZN44 BtCoV/HKU952
A3EXH4 BtCoV/HKU92
A3EXJ0 BtCoV/HKU94
A0A1B3Q5W5 BtCoV/GCCDC1 356
A3EXD0 BtCoV/HKU5/2004
S4WWS3 BtCoV/HKU5/2004
A0A0U1WHM0 BtCoV/GD2013
Q0Q4F7 BtCoV/A434/2005
A3EXD9 BtCoV/HKU52
A3EXE8 BtCoV/HKU53
A3EXF7 BtCoV/HKU55
S4X422 BtCoV/HKU5/2004
A0A5H2WTJ3 BtCoV/VsCoV1
A0A2I6PIX8 BtCoV//P.khulii/Italy/20664563/2011
A0A2I4R8V9 BtCoV/HKU25
A0A2I4R888 BtCoV/HKU25
A0A166ZLN4 BtCoV/JPDB144
A0A0U1WJZ6 BtCoV/GX2012
Q0Q4F2 BtCoV/133/2005
A0A1Z1W244 BtCoV/161028
A0A1Z1W227 BtCoV/160079
A0A1Z1W230 BtCoV/160081
S4WYD2 BtCoV/HKU4/2004
A3EXC1 BtCoV/HKU44
S4WWQ3 BtCoV/HKU4/2004
S4WYD6 BtCoV/HKU4/2004
S4WZQ1 BtCoV/HKU4/2004
A3EXA3 BtCoV/HKU42
A3EX94 BtCoV/HKU4/2004
A3EXB2 BtCoV/HKU43
A0A2R4KP93 BtMERSrCoV/NL140422
A0A2R4KP86 BtMERSrCoV/NL13845
A0A0A0Q7F3 MERSCoV
A0A1W6ASU7 BtCoV/PREDICT/PDF2180
U5NJG5 BtCoV/Neoromicia/PMLPHE1/RSA/2011
R9QTA0 BtCoV/Rp/Shaanxi2011
Q0QDX9 BtCoV/Rm1/2004
Q0Q475 BtCoV/279/2005
Q3I5J5 BtCoV/Rp3/2004
A0A1W6S788 BtCoV/F46
A0A2D1PX37 BtCoV/Rf4092
A0A2R3SUW9 BtCoV/ZXC21
A0A2R3SUW7 BtCoV/ZC45
A0A0K1Z054 BtCoV/YNLF 34C
A0A0K1Z074 BtCoV/YNLF 31C
Q0Q484 BtCoV/273/2005
Q0QDZ0 BtCoV/Rf1/2004
A0A0U1WHL1 BtCoV/HuB2013
A0A0U1WHI6 BtCoV/SX2013
A0A0U1WHK9 BtCoV/HeN2013
A0A1W5YKU9 BtCoV/Jiyuan84
A0A0U1WHH0 BtCoV/HeB2013
A0A166ZL64 BtCoV/JTMC15
A0A0U1UYX4 BtCoV/JL2012
A0A221ZS09 BtCoV/16BO133
A0A173G2Q3 BtCoV/B1521
A0A1W5YKT9 BtCoV/Anlong103
A0A0U1WJY8 BtCoV/YN2013
A0A4Y6GL43 BtCoV/SC2018
A0A0U1WHI2 BtCoV/HuB2013
A0A4Y6GL75 BtCoV/YN2018A
A0A2D1PX86 BtCoV/Rs4247
D5HJU5 BtCoV/HKU38
D5HJT4 BtCoV/HKU37
A0A0U1WHJ8 BtCoV/GX2013
Q3LZV3 BtCoV/HKU32
Q3LZX1 SARSrBtCoV/HKU3
Q3LZT7 BtCoV/HKU33
D5HK02 BtCoV/HKU313
D5HJZ1 BtCoV/HKU312
D5HJW9 BtCoV/HKU310
D5HJV8 BtCoV/HKU39
D5HJS3 BtCoV/HKU36
D5HJR2 BtCoV/HKU35
D5HJQ1 BtCoV/HKU34
A0A096XNM6 BtCoV/Longquan140
A0A2D1PX73 BtCoV/Rs4237
A0A2D1PX05 BtCoV/As6526
A0A4Y6GL82 BtCoV/YN2018C
A0A2D1PX88 BtCoV/Rs4255
A0A4Y6GL56 BtCoV/YN2018D
A0A2D1PX44 BtCoV/Rs4081
A0A166ZND9 BtCoV/MLHJC35
R9QTH3 BtCoV/Cp/Yunnan2011
A0A6M3G9R1 PCoV GD MP789
P0DTC2 SARSCoV2
A0A6B9WHD3 BtCoV/RaTG13
A0A6G6A2R8 PCoV GXP1E
A0A6G6A1M4 PCoV GXP5L
A0A6G6A2Q2 PCoV GXP4L
A0A6G9KP06 PCoV GXP2V
A0A6G6A331 PCoV GXP5E
P59594 SARSCoV
A0A2D1PXC0 BtCoV/Rs7327
A0A4Y6GL47 BtCoV/YN2018B
A0A2D1PXD5 BtCoV/Rs9401
A0A0U2IWM2 BtCoV/WIV16
A0A2D1PX97 BtCoV/Rs4874
U5WHZ7 BtCoV/Rs3367
U5WI05 BtCoV/WIV1
A0A2D1PX29 BtCoV/Rs4084
U5WLK5 BtCoV/RsSHC014
A0A2D1PXA9 BtCoV/Rs4231
E0XIZ3 BtCoV/BM4831/BGR/2008
F1DAZ1 BtCoV/Kenya/KY24/2006
A0A2Z4EVN2 BtCoV/CMR891892
A0A2Z4EVR7 BtCoV/CMR705P13
A0A2Z4EVK1 BtCoV/CMR704P12
A0A2Z4EVN5 BtCoV/CMR900
F1DAZ9 BtCoV/Kenya/KY06/2006
A0A2Z4EVM5 BtCoV/CMR66
A3EXG6 BtCoV/HKU9
A0A2P1M5J5 BtCoV/HKU9
A0A4Y6GL90 BtCoV/GX2018
A0A5B9Y142 BtCoV/BtCoV92
A3EXI2 BtCoV/HKU93
E0ZN36 BtCoV/HKU951
E0ZN52 BtCoV/HKU9101
E0ZN60 BtCoV/HKU9102
E0ZN44 BtCoV/HKU952
A3EXH4 BtCoV/HKU92
A3EXJ0 BtCoV/HKU94
A0A1B3Q5W5 BtCoV/GCCDC1 356
A3EXD0 BtCoV/HKU5/2004
S4WWS3 BtCoV/HKU5/2004
A0A0U1WHM0 BtCoV/GD2013
Q0Q4F7 BtCoV/A434/2005
A0A5H2WTJ3 BtCoV/VsCoV1
A3EXD9 BtCoV/HKU52
A3EXF7 BtCoV/HKU55
A3EXE8 BtCoV/HKU53
S4X422 BtCoV/HKU5/2004
A0A2I6PIX8 BtCoV//P.khulii/Italy/20664563/2011
A0A2I4R8V9 BtCoV/HKU25
A0A2I4R888 BtCoV/HKU25
A0A166ZLN4 BtCoV/JPDB144
A0A0U1WJZ6 BtCoV/GX2012
Q0Q4F2 BtCoV/133/2005
A0A1Z1W244 BtCoV/161028
A0A1Z1W227 BtCoV/160079
S4WYD2 BtCoV/HKU4/2004
A0A1Z1W230 BtCoV/160081
A3EXC1 BtCoV/HKU44
S4WWQ3 BtCoV/HKU4/2004
S4WZQ1 BtCoV/HKU4/2004
S4WYD6 BtCoV/HKU4/2004
A3EXA3 BtCoV/HKU42
A3EX94 BtCoV/HKU4/2004
A3EXB2 BtCoV/HKU43
A0A2R4KP93 BtMERSrCoV/NL140422
A0A2R4KP86 BtMERSrCoV/NL13845
A0A0A0Q7F3 MERSCoV
A0A1W6ASU7 BtCoV/PREDICT/PDF2180
U5NJG5 BtCoV/Neoromicia/PMLPHE1/RSA/2011
A0A1W6S788 BtCoV/F46
A0A2D1PX37 BtCoV/Rf4092
A0A2R3SUW9 BtCoV/ZXC21
A0A2R3SUW7 BtCoV/ZC45
A0A0K1Z054 BtCoV/YNLF 34C
A0A0K1Z074 BtCoV/YNLF 31C
Q0QDZ0 BtCoV/Rf1/2004
Q0Q484 BtCoV/273/2005
A0A0U1WHI6 BtCoV/SX2013
A0A0U1WHK9 BtCoV/HeN2013
A0A0U1WHH0 BtCoV/HeB2013
A0A1W5YKU9 BtCoV/Jiyuan84
A0A0U1WHL1 BtCoV/HuB2013
A0A166ZL64 BtCoV/JTMC15
A0A221ZS09 BtCoV/16BO133
A0A0U1UYX4 BtCoV/JL2012
A0A173G2Q3 BtCoV/B1521
A0A1W5YKT9 BtCoV/Anlong103
A0A0U1WJY8 BtCoV/YN2013
R9QTA0 BtCoV/Rp/Shaanxi2011
Q0QDX9 BtCoV/Rm1/2004
Q0Q475 BtCoV/279/2005
Q3I5J5 BtCoV/Rp3/2004
A0A2D1PX73 BtCoV/Rs4237
A0A2D1PX88 BtCoV/Rs4255
A0A2D1PX05 BtCoV/As6526
A0A4Y6GL82 BtCoV/YN2018C
A0A4Y6GL56 BtCoV/YN2018D
A0A2D1PX44 BtCoV/Rs4081
A0A166ZND9 BtCoV/MLHJC35
R9QTH3 BtCoV/Cp/Yunnan2011
A0A0U1WHI2 BtCoV/HuB2013
A0A4Y6GL43 BtCoV/SC2018
D5HJU5 BtCoV/HKU38
D5HJT4 BtCoV/HKU37
A0A2D1PX86 BtCoV/Rs4247
A0A4Y6GL75 BtCoV/YN2018A
A0A0U1WHJ8 BtCoV/GX2013
Q3LZV3 BtCoV/HKU32
Q3LZX1 SARSrBtCoV/HKU3
D5HK02 BtCoV/HKU313
Q3LZT7 BtCoV/HKU33
D5HJW9 BtCoV/HKU310
D5HJS3 BtCoV/HKU36
D5HJQ1 BtCoV/HKU34
D5HJZ1 BtCoV/HKU312
D5HJR2 BtCoV/HKU35
D5HJV8 BtCoV/HKU39
A0A096XNM6 BtCoV/Longquan140
A0A6G6A2R8 PCoV GXP1E
A0A6M3G9R1 PCoV GD MP789
P0DTC2 SARSCoV2
A0A6B9WHD3 BtCoV/RaTG13
A0A6G6A1M4 PCoV GXP5L
A0A6G6A2Q2 PCoV GXP4L
A0A6G6A331 PCoV GXP5E
A0A6G9KP06 PCoV GXP2V
P59594 SARSCoV
A0A2D1PXC0 BtCoV/Rs7327
A0A4Y6GL47 BtCoV/YN2018B
A0A2D1PXD5 BtCoV/Rs9401
A0A0U2IWM2 BtCoV/WIV16
A0A2D1PX97 BtCoV/Rs4874
U5WHZ7 BtCoV/Rs3367
U5WI05 BtCoV/WIV1
A0A2D1PX29 BtCoV/Rs4084
U5WLK5 BtCoV/RsSHC014
A0A2D1PXA9 BtCoV/Rs4231
E0XIZ3 BtCoV/BM4831/BGR/2008
No ACE2 Binding
Binds ACE2
Non−Human−Host CoV
Human−Host CoV

S8:RBD ML (L) vs 3 Euc UP (R)

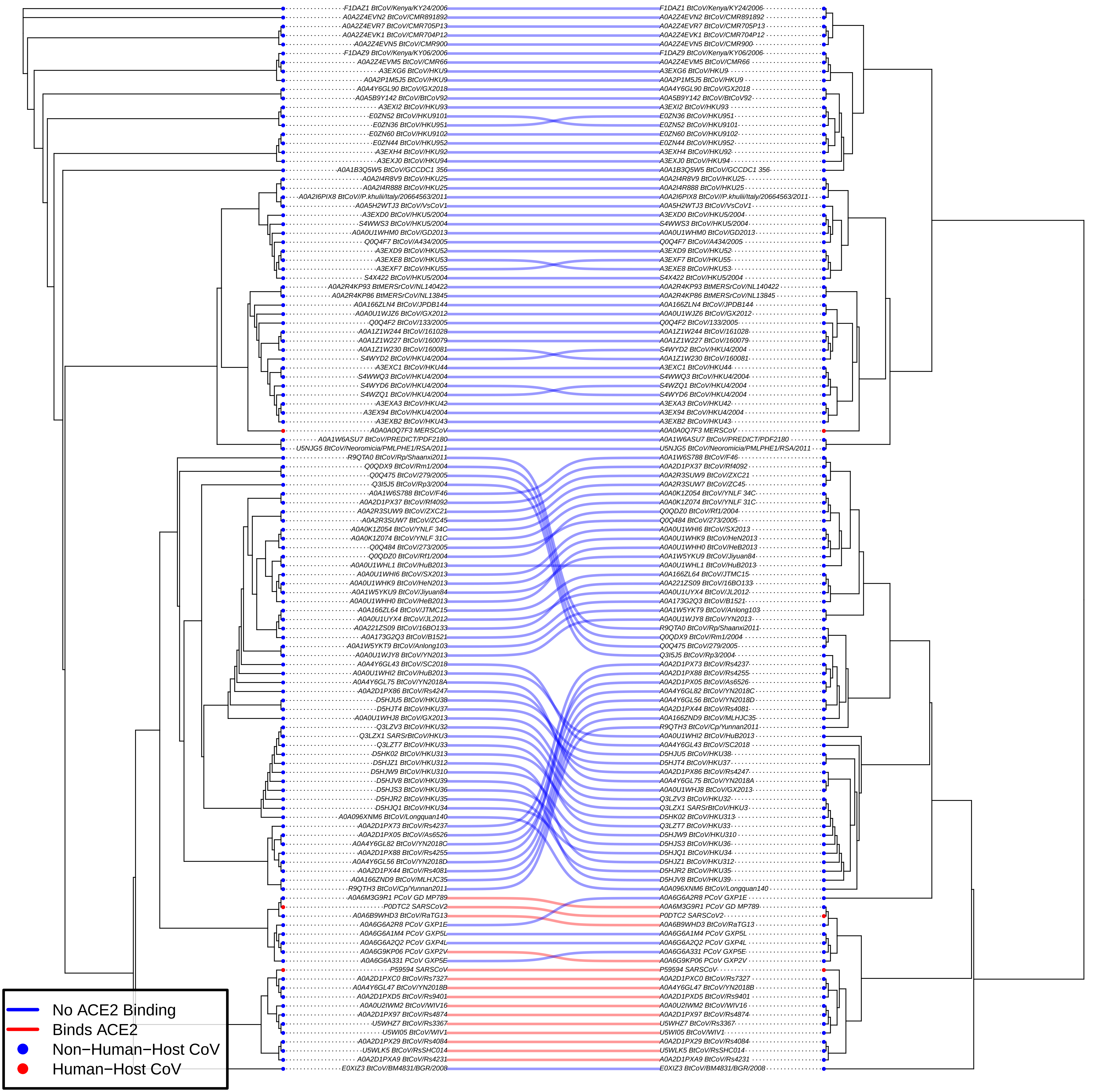

S9:RBD ML (L) vs 3 Cos NJ (R)

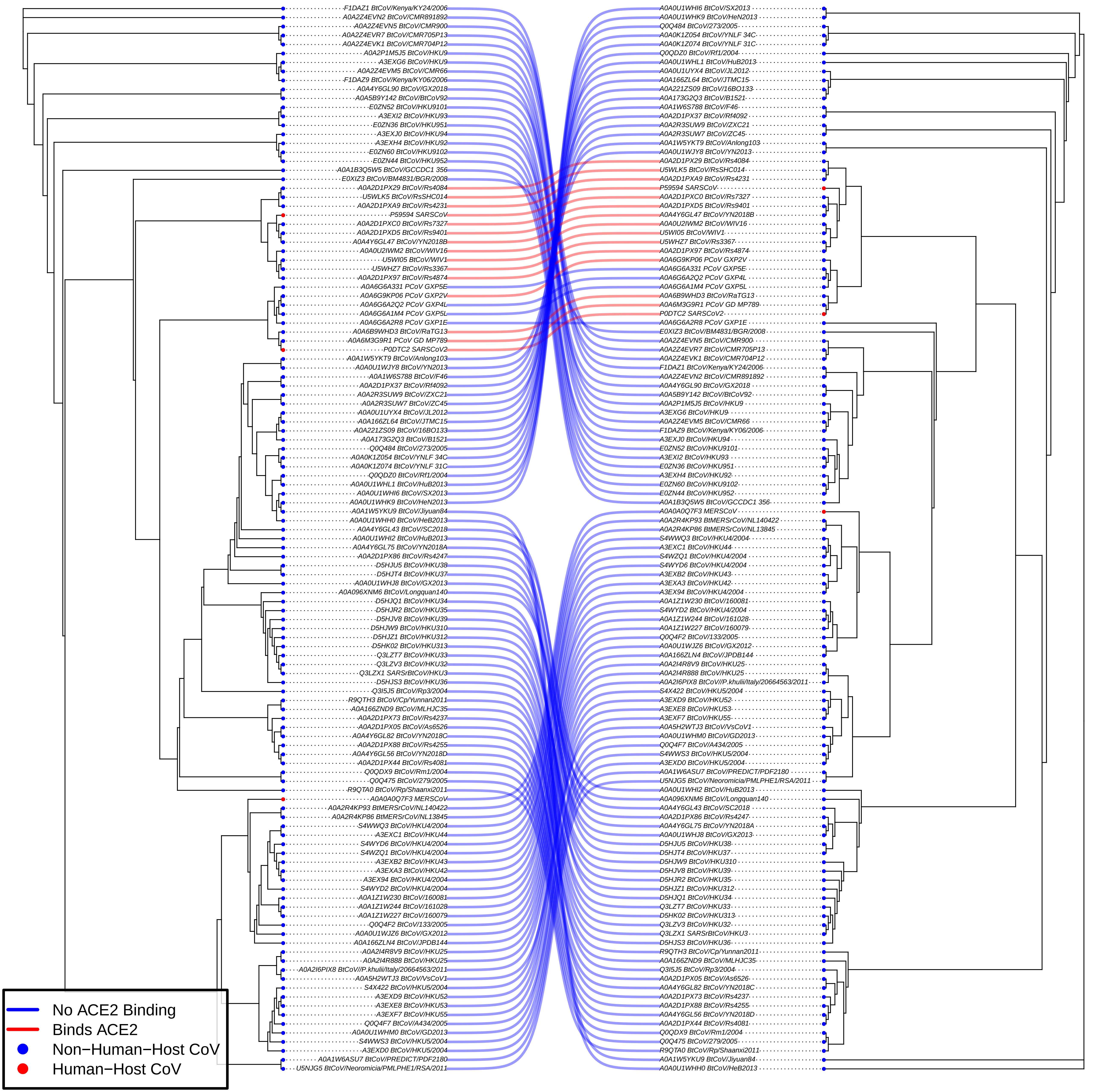

S10:RBD ML (L) vs 3 Euc NJ (R)

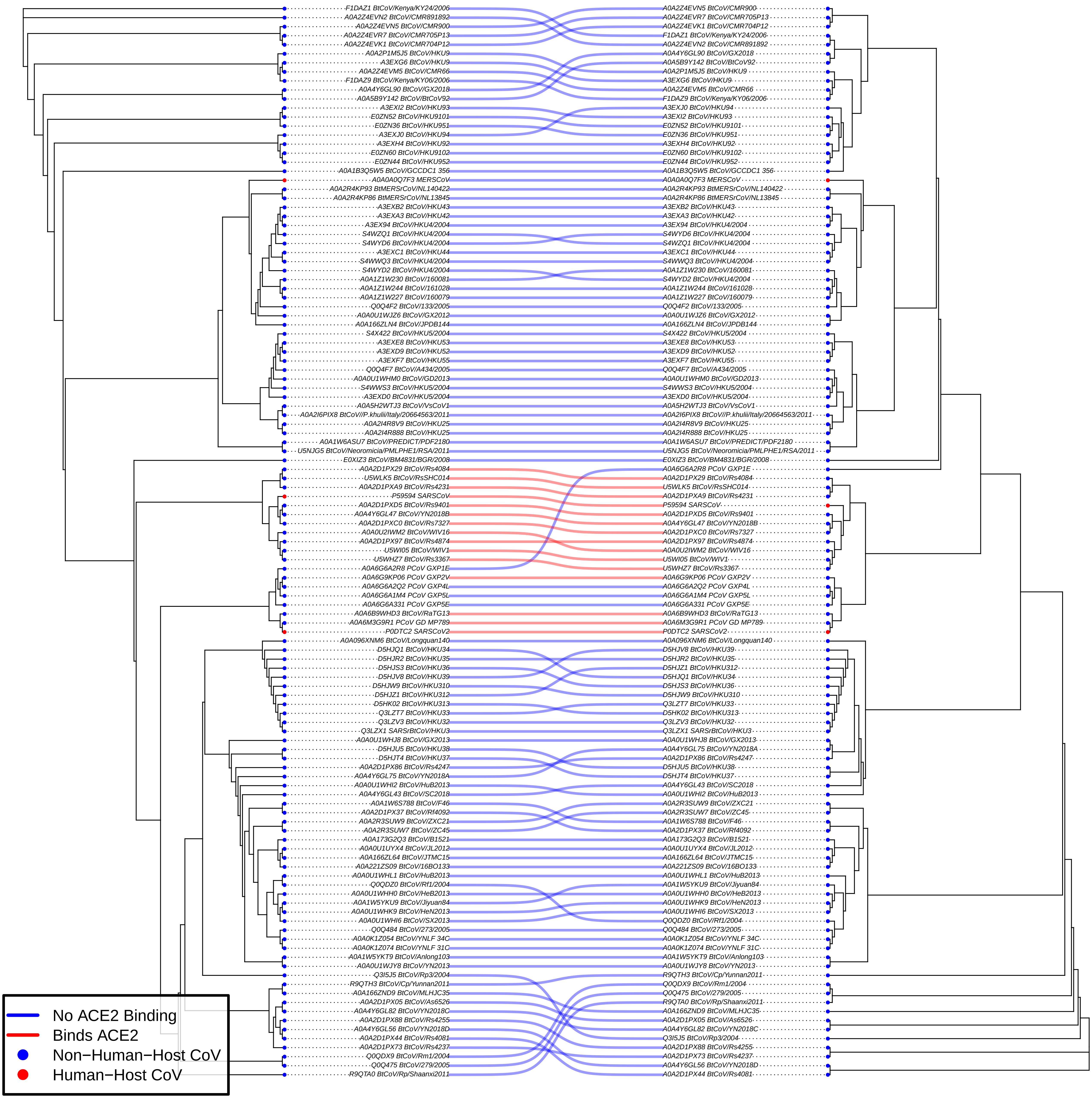

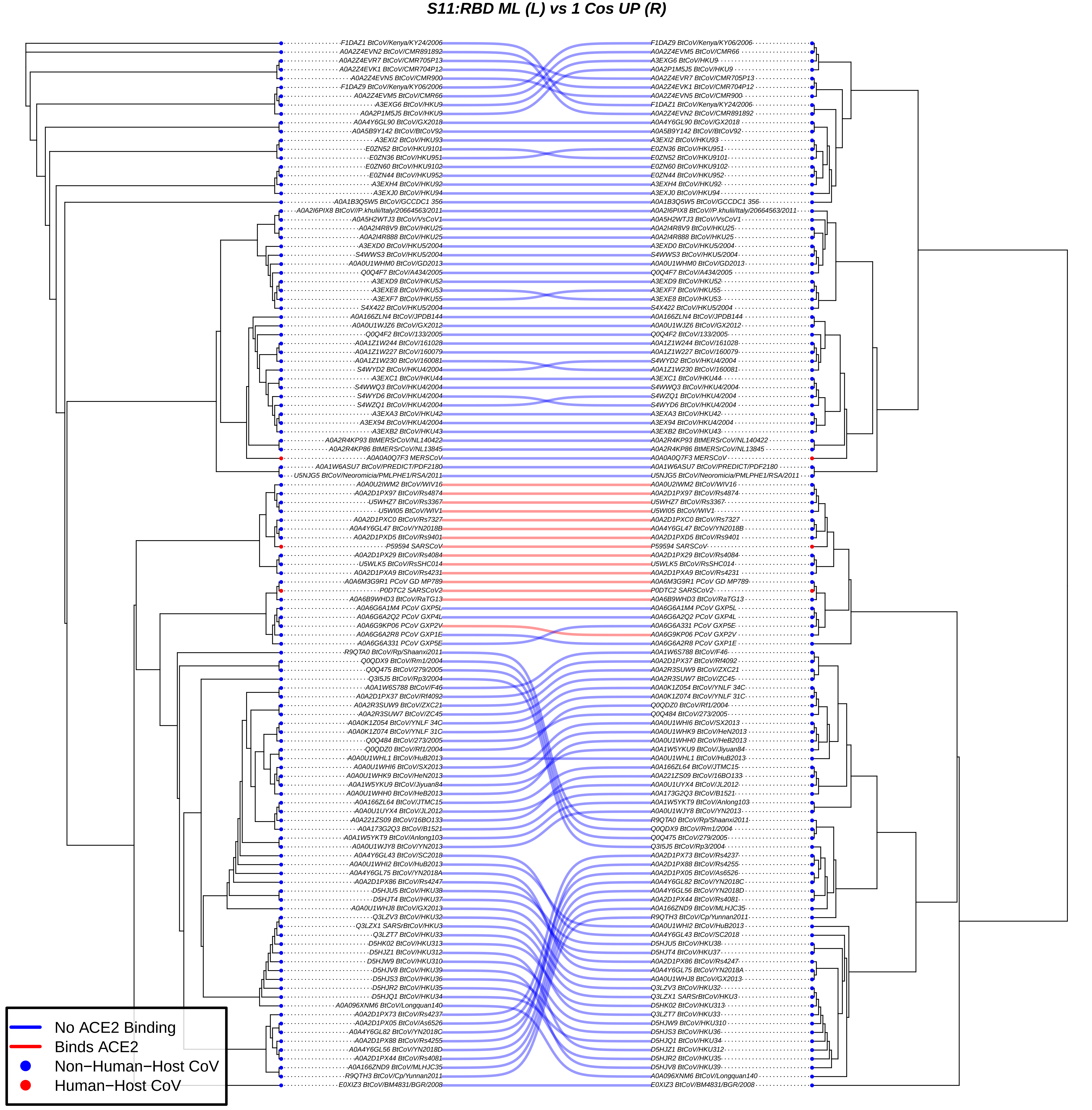
S11:RBD ML (L) vs 1 Cos UP (R)
No ACE2 Binding
Binds ACE2
Non−Human−Host CoV
Human−Host CoV

S12:RBD ML (L) vs 1 Euc UP (R)

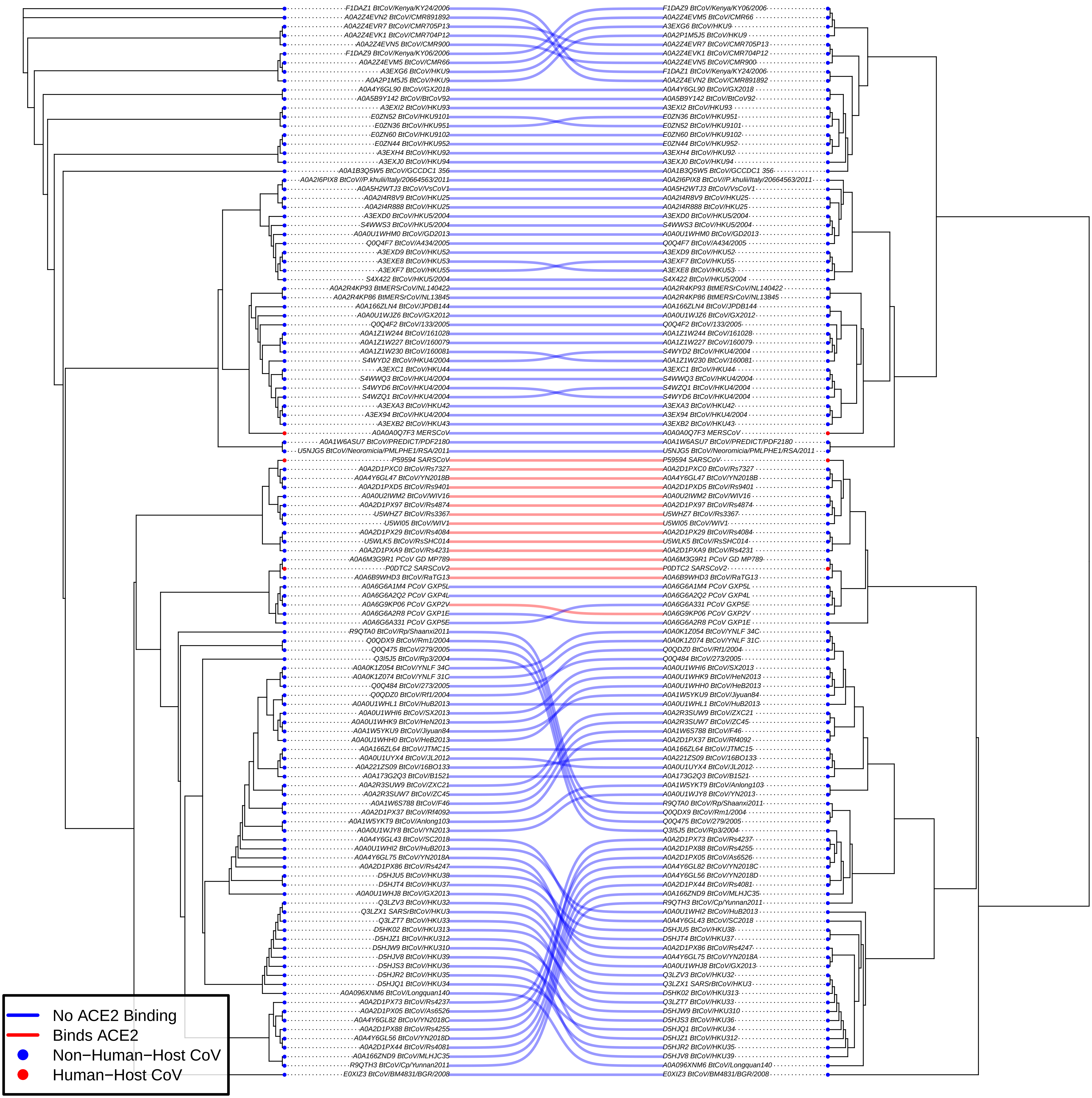

S13:RBD ML (L) vs 1 Cos NJ (R)

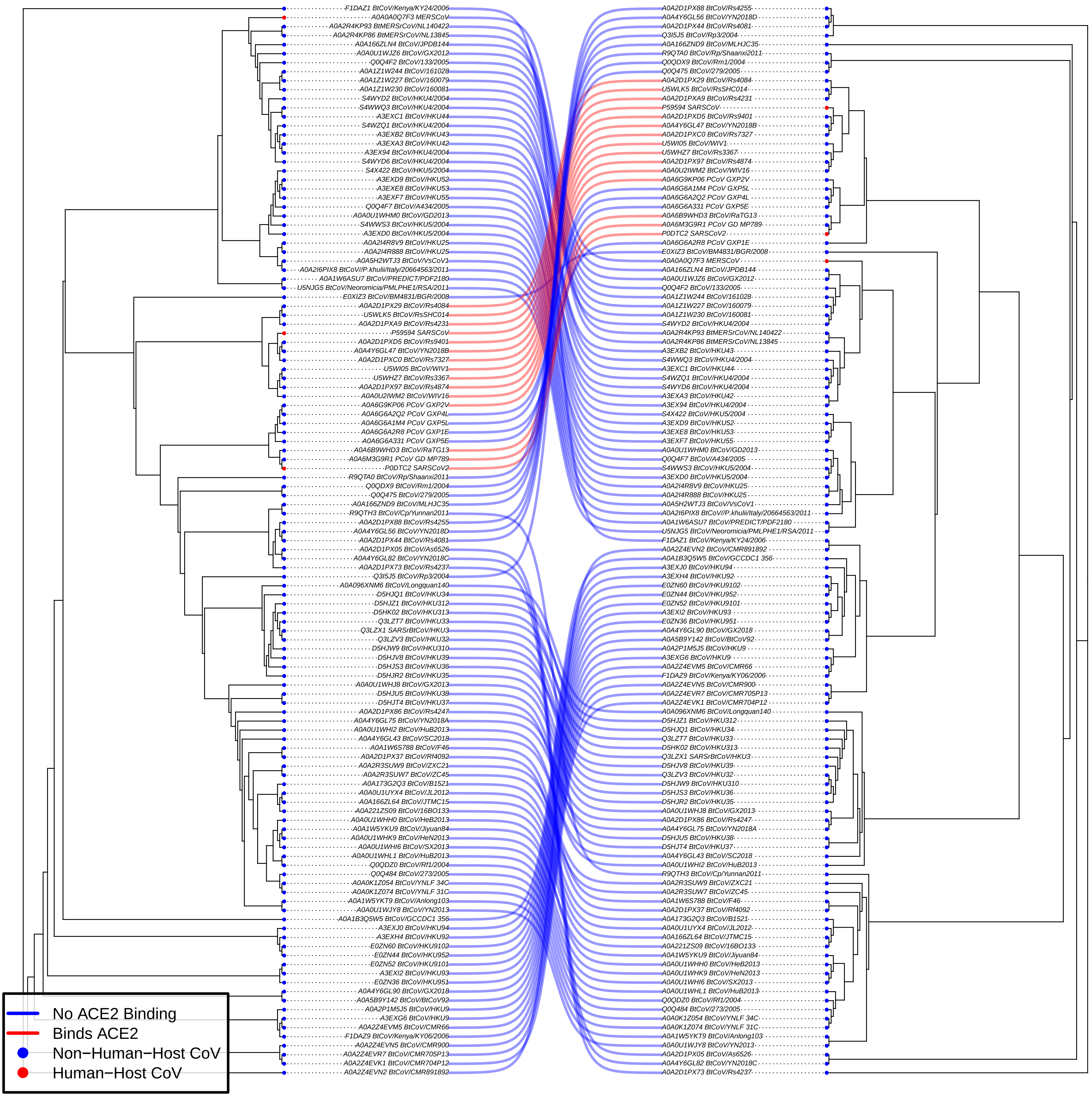

S14:RBD ML (L) vs 1 Euc NJ (R)

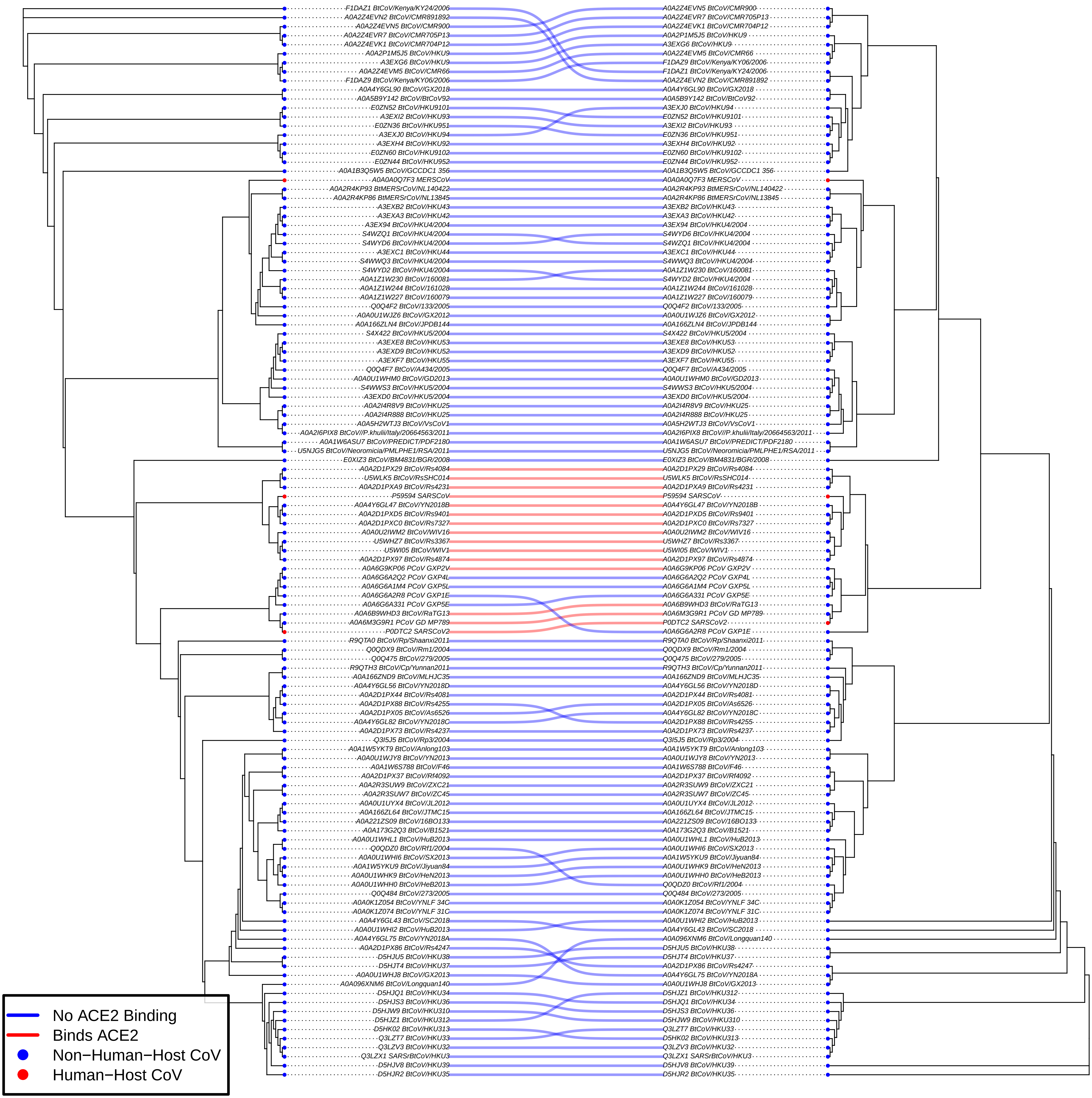

Supplement: eoab032_Supplementary_Data [file eoab032_supplementary_data.zip › Supplemental Figures.pdf]
